# Targeted dephosphorylation of SMAD3 as an approach to impede TGF-β signaling

**DOI:** 10.1016/j.isci.2024.110423

**Published:** 2024-07-05

**Authors:** Abigail Brewer, Jin-Feng Zhao, Rotimi Fasimoye, Natalia Shpiro, Thomas J. Macartney, Nicola T. Wood, Melanie Wightman, Dario R. Alessi, Gopal P. Sapkota

**Affiliations:** 1 Medical Research Council (MRC) Protein Phosphorylation & Ubiquitylation Unit, School of Life Sciences, University of Dundee, Dundee DD1 5EH, UK

**Keywords:** Biochemistry, Biochemistry methods, Cell biology

## Abstract

TGF-β (transforming growth factor-β) signaling is involved in a myriad of cellular processes and its dysregulation has been implicated in many human diseases, including fibrosis and cancer. TGF-β transcriptional responses are controlled by tail phosphorylation of transcription factors SMAD2 and SMAD3 (mothers against decapentaplegic homolog 2/3). Therefore, targeted dephosphorylation of phospho-SMAD3 could provide an innovative mechanism to block some TGF-β-induced transcriptional responses, such as the transcription of *SERPINE-1*, which encodes plasminogen activator inhibitor 1 (PAI-1). Here, by developing and employing a bifunctional molecule, BDPIC (bromoTAG-dTAG proximity-inducing chimera), we redirected multiple phosphatases, tagged with bromoTAG, to dephosphorylate phospho-SMAD3, tagged with dTAG. Using CRISPR-Cas9 technology, we generated homozygous double knock-in A549 ^*bromoTAG/bromoTAG*^*PPM1H/*^*dTAG/dTAG*^*SMAD3* cells, in which the BDPIC-induced proximity between bromoTAG-PPM1H and dTAG-SMAD3 led to a robust dephosphorylation of dTAG-SMAD3 and a significant decrease in *SERPINE-1* transcription. Our work demonstrates targeted dephosphorylation of phospho-proteins as an exciting modality for rewiring cell signaling.

## Highlights

• Generated a bifunctional molecule, BDPIC (bromoTAG-dTAG proximity-inducing chimera)
• BDPIC recruits bromoTAG-PPM1H to dephosphorylate dTAG-SMAD3 tail residues in cells
• Targeted dephosphorylation of dTAG-SMAD3 impedes downstream TGF-β signaling
• TGF-β-induced EMT is impeded by targeted dTAG-SMAD3 dephosphorylation

## Graphical abstract

**Figure:**
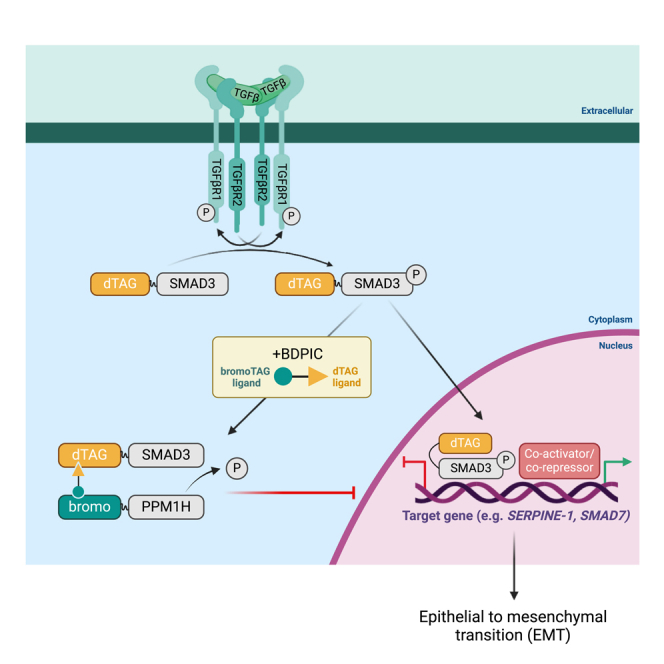

## Introduction

TGF-β (transforming growth factor-β) is a cytokine implicated in a plethora of cellular processes, such as proliferation, regeneration, differentiation, adhesion, cell migration, and apoptosis. Canonical TGF-β signaling involves binding of the TGF-β ligand to a constitutively active transmembrane Ser/Thr kinase receptor, TGF-βRII (type II receptor), which then recruits, phosphorylates, and activates TGF-βRI (type I receptor). Transcription factors SMAD2 and SMAD3 (mothers against decapentaplegic homolog 2/3) are recruited to the active receptor complex. SMAD2 and SMAD3 comprise a variable linker region that separates two conserved domains: MH1 (MAD homology 1), which modulates DNA binding, and MH2 (MAD homology 2), which mediates protein-protein interactions, such as with TGF-βRI and transcriptional co-activators/-repressors.^1^^,^^2^^,^^3^ Once at the receptor complex, SMAD2 and SMAD3 are phosphorylated by TGF-βRI at a C-terminal tail Ser-X-Ser motif (Ser465/Ser467 on SMAD2 and Ser423/Ser425 on SMAD3). As such, several small molecule inhibitors of TGF-βRI, including SB-505124, result in complete attenuation of TGF-β-induced SMAD2/3 tail phosphorylation and downstream signaling.^4^^,^^5^ Phosphorylated SMAD2 and SMAD3 are released from the receptor complex and bind to co-SMAD, SMAD4, before translocating to the nucleus, where they control the transcription of hundreds of target genes that dictate cell fate decisions.^6^ Despite sharing 83.9% amino acid sequence, SMAD2 and SMAD3 are believed to possess distinct cellular roles. It is thought that most TGF-β-induced transcriptional responses in adult tissues and cells, which can activate cytostatic and apoptotic responses, are mediated by SMAD3, while SMAD2 is essential during development, where TGF-β signaling promotes growth and differentiation.^7^^,^^8^^,^^9^^,^^10^^,^^11^

TGF-β signaling has been implicated in numerous pathologies and previous studies have identified SMAD3 as a key contributor to the progression of some TGF-β-associated diseases.^12^^,^^13^ Gain-of-function *SMAD3* mutation, which leads to increased TGF-β signaling, has been linked to melorheostosis, a rare sporadic disorder involving excessive bone formation on the bone surface.^14^^,^^15^ Furthermore, increased TGF-β pathway activation and subsequent SMAD3 phosphorylation have also been implicated in fibrotic diseases through promotion of influx of inflammatory cells and fibroblasts to the site of injury, followed by production of cytokines and ECM (extracellular matrix), respectively.^16^ Accumulation of ECM is then further promoted by TGF-β through simultaneously increasing secretion of protease inhibitors (such as SMAD3-dependent PAI-1 (plasminogen activator inhibitor 1)) and reducing secretion of proteases (such as MMP-1 (matrix metalloproteinase-1)).^17^^,^^18^^,^^19^ Fibrotic disease is thought to arise following incomplete resolution of tissue repair. Dysregulation of TGF-β signaling is also thought to trigger a shift from normal pathway function, which is considered to perform a cytostatic and tumor suppressor role, to oncogenic function.^11^ This shift occurs particularly in the advanced stages of cancer and can be driven by SMAD3.^11^ In addition to tail phosphorylation, SMAD3 transcriptional activity is also regulated by phosphorylation of the linker region, which can occur at multiple sites, including Thr179, Ser204, and Ser208, by various kinases, such as MAPKs (mitogen-activated protein kinases), CDKs (cyclin-dependent kinases), and GSK3β (glycogen synthase kinase-3β).^20^^,^^21^ Alterations in SMAD3 linker phosphorylation are believed to contribute to tumor progression by promoting EMT (epithelial to mesenchymal transition) of cells.^21^ Given that SMAD3 function is controlled by phosphorylation, targeted dephosphorylation of SMAD3 could potentially afford inhibition of some TGF-β signaling responses, which may be of therapeutic benefit.

Targeted dephosphorylation of phospho-proteins is an emerging strategy to modulate protein function and cell signaling with substrate-level phospho-control. Redirecting phosphatase activity to elicit targeted dephosphorylation of a POI (protein of interest) to alter POI function could provide a promising innovative therapeutic mechanism for combatting pathologies in which POI phosphorylation is implicated. Furthermore, substrate-specific dephosphorylation may overcome some of the limitations currently experienced with kinase inhibitors, which can attenuate the phosphorylation of other kinase substrates in addition to the desired substrate.^22^ We previously reported the development of the AdPhosphatase (affinity-directed phosphatase) system, which uses polypeptide binders to deliver phosphatase activity to phospho-POIs to elicit targeted POI dephosphorylation.^23^ Additional recent studies have also explored targeted protein dephosphorylation. Bifunctional molecules comprising a HaloTag or peptidic ligand to bind protein phosphatase PP1 and selective inhibitors to bind Akt or EGFR (epidermal growth factor receptor) were shown to reduce Akt and EGFR phosphorylation.^24^ Another study reported development of a bifunctional recruiter of dTAG and HaloTag, termed PhosTAC7 (phosphorylation-targeting chimera), to recruit dTAG-PP2A A (the scaffolding A subunit of protein phosphatase 2A) to Halo-PDCD4 (programmed cell death 4),^25^ Halo-FOXO3a (forkhead box O3),^25^ and Halo-Tau^26^ to decrease phosphorylation of the Halo-POIs. More recently, it was shown that a bifunctional molecule that recruits endogenous PP5 to ASK1 (apoptosis signal-regulated kinase 1) decreased ASK1 phosphorylation and concomitantly displayed antiproliferative activity in gastric cancer cells.^27^ These studies using bifunctional molecules have demonstrated the exciting potential of targeted protein dephosphorylation, however, most have relied on overexpression model systems and sub-optimal compounds (e.g., requiring high micromolar concentrations).

In this study, we developed a bifunctional molecule, which we named BDPIC (bromoTAG-dTAG proximity-inducing chimera) and employed it at nanomolar concentrations to screen different phosphatases tagged with bromoTAG for targeted dephosphorylation of phospho-SMAD3 tagged with dTAG. We found that bromoTAG-PPM1H phosphatase activity can be redirected to dephosphorylate TGF-β-induced phospho-dTAG-SMAD3 in both stable expression and endogenous model systems, and we investigated subsequent inhibition of some TGF-β-transcriptional responses.

## Results

### Development and characterization of BDPIC

**Figure 1:**
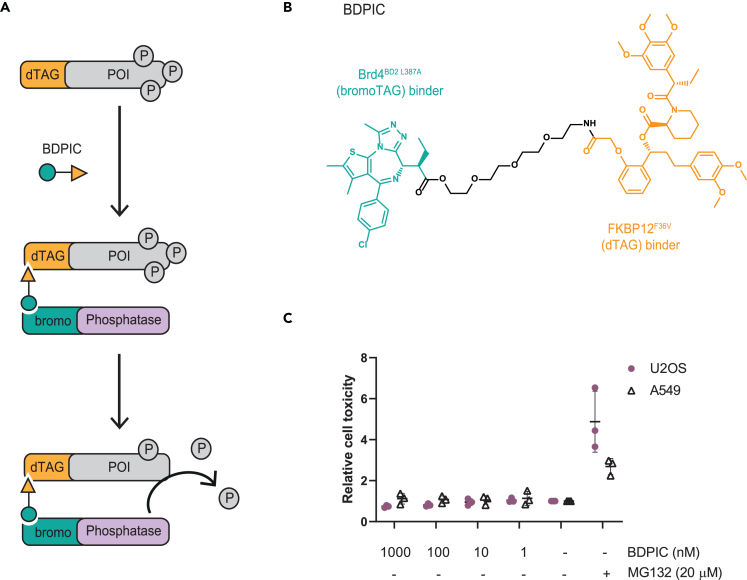
Design and characterization of BDPIC (bromoTAG-dTAG proximity-inducing chimera) (A) Schematic representation of BDPIC-mediated induction of proximity between a bromoTAG-phosphatase and a dTAG-POI to elicit targeted dephosphorylation of the dTAG-POI. The extent of dephosphorylation of a multi-phosphorylated dTAG-POI would likely depend on the specific phosphatase-POI pair. (B) Structure of BDPIC with the bromoTAG- and dTAG-binding moieties highlighted. (C) Cytotoxicity of BDPIC was measured using CellTox Green Assay (Promega) by treating wild-type (WT) U2OS or A549 cells with BDPIC at the indicated concentrations for 24 h, with DMSO employed as a negative control and MG132 (20 μM, 24 h) as a positive control for cytotoxicity. Fluorescence was measured using a PHERAstar plate reader (ex: 480 nm em: 530 nm) and data normalized to background. Data represent *n* = 3. Values are shown as a mean fluorescence reading normalized to DMSO controls ±SD.

To probe whether chemically inducing proximity to a phosphatase could be sufficient to enable targeted dephosphorylation of a POI, we conceptualized that by linking the established bromoTAG and dTAG ligands,^28^^,^^29^^,^^30^^,^^31^^,^^32^^,^^33^^,^^34^^,^^35^ the resulting bifunctional molecule could induce proximity between bromoTAG and dTAG in cells. We sought to use this proof-of-concept bifunctional molecule to recruit a bromoTAG-phosphatase to a dTAG-POI to target the dephosphorylation of one or more dTAG-POI phospho-sites (Figure 1A). Opting for a 3 PEG (polyethylene glycol) linker, we synthesized the compound, which we named BDPIC (bromoTAG-dTAG proximity-inducing chimera) (Figure 1B) and confirmed that BDPIC did not cause cytotoxicity in U2OS or A549 cells upon exposure to a maximum of 1 μM BDPIC for 24 h (Figure 1C). Used as a positive control, MG132 caused profound cell death in both U2OS and A549 cells compared to DMSO control treatment (Figure 1C).

### BDPIC can mediate targeted dephosphorylation of dTAG-SMAD3 through an induced proximity to bromoTAG-phosphatases

**Figure 2:**
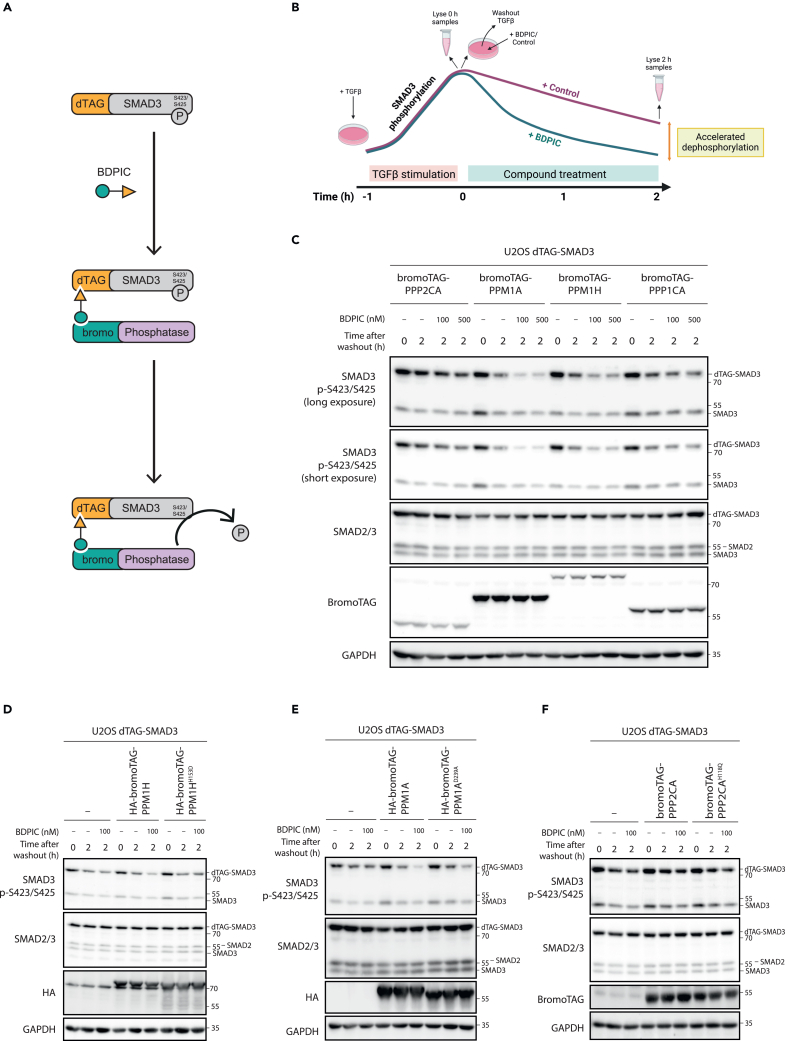
Targeted dephosphorylation of dTAG-SMAD3 through BDPIC-mediated recruitment of bromoTAG-phosphatases (A) Schematic representation of BDPIC-mediated induction of proximity between a bromoTAG-phosphatase and dTAG-SMAD3 to elicit targeted dephosphorylation of dTAG-SMAD3. (B) Schematic of the washout style assay designed to monitor rate of dTAG-SMAD3 dephosphorylation upon BDPIC treatment. Cells are stimulated for 1 h with TGF-β to establish maximal SMAD3 phosphorylation. At this point, cells are either lysed to capture maximal SMAD3 phosphorylation (0 h) or the TGF-β stimulation is washed out and cells are treated with either BDPIC or DMSO control for 2 h prior to lysis. DMSO control reveals the natural rate of SMAD3 dephosphorylation while targeted dephosphorylation by BDPIC would be expected to cause an accelerated rate of dTAG-SMAD3 dephosphorylation when measured at 2 h. Schematic was generated using BioRender. (C) Screening of BDPIC-mediated recruitment of different bromoTAG-phosphatases to target dTAG-SMAD3 dephosphorylation. U2OS cells were retrovirally transduced to co-express dTAG-SMAD3 and the indicated bromoTAG-phosphatases. Cells were serum-starved (16 h) prior to stimulation with TGF-β (1 h, 5 μg/L). 0 h time points were lysed at this moment. For 2 h time points, TGF-β stimulation was removed by washout and fresh serum-free medium without TGF-β was added to cells, along with DMSO or BDPIC treatment at the indicated concentrations for 2 h. Cells were then lysed before extracts were resolved by SDS-PAGE, transferred to nitrocellulose membrane and subjected to immunoblotting with the indicated antibodies. (D) As in (C) except here U2OS cells expressing dTAG-SMAD3 alone or in combination with bromoTAG-PPM1H or catalytically inactive bromoTAG-PPM1H^H153D^ were used. (E) As in (C) except here U2OS cells expressing dTAG-SMAD3 alone or in combination with bromoTAG-PPM1A or catalytically inactive bromoTAG-PPM1A^D239A^ were used. (F) As in (C) except here U2OS cells expressing dTAG-SMAD3 alone or in combination with bromoTAG-PPP2CA or catalytically inactive bromoTAG-PPP2CA^H118Q^ were used. Data are representative of *n* = 3 independent experiments.

We sought to assess the ability of BDPIC to induce dephosphorylation of dTAG-SMAD3 in cells co-expressing a bromoTAG-phosphatase (Figure 2A). As a first step, the physiological rate of dTAG-SMAD3 phosphorylation upon stimulation with recombinant TGF-β ligand was characterized over 6 h in serum-starved U2OS cells stably expressing dTAG-SMAD3 (Figures S1A and S1B). Maximal tail phosphorylation of dTAG-SMAD3 and endogenous SMAD3 was observed following 1 h stimulation, with phosphorylation decreasing over the remaining treatment durations, likely due to receptor turnover,^36^ but remaining higher than in unstimulated cells (Figures S1A and S1B). Importantly, this indicates that conjugation of dTAG to SMAD3 has not impeded SMAD3 phosphorylation by TGF-βRI. Increased protein levels of PAI-1, whose gene transcription is SMAD3-dependent, were detected as soon as 2 h after initiation of TGF-β stimulation (Figures S1A and S1B). Next, we monitored the physiological rate of dTAG-SMAD3 dephosphorylation in serum-starved cells. Following 1 h stimulation with TGF-β, U2OS cells stably expressing dTAG-SMAD3 were either lysed (termed 0 min after washout) or washed with PBS (phosphate-buffered saline) and placed in fresh serum-free medium without TGF-β and lysed at the specified time intervals (Figures S1C and S1D). In comparison to unstimulated cells, TGF-β-treated cells lysed 0 min after washout displayed a marked increase in dTAG-SMAD3 and endogenous SMAD3 tail phosphorylation. A reduction in dTAG-SMAD3 phosphorylation was detected as soon as 10 min following TGF-β washout, with ∼60% reduction in phosphorylation apparent by 120 min and ∼80% by 360 min (Figures S1C and S1D).

For our targeted dephosphorylation approach in which we aimed to employ BDPIC to recruit a bromoTAG-phosphatase to dephosphorylate dTAG-SMAD3, we considered that co-treatment of TGF-β (to establish dTAG-SMAD3 phosphorylation) and BDPIC could potentially block dTAG-SMAD3 phosphorylation via BDPIC-recruited bromoTAG-phosphatase limiting access of dTAG-SMAD3 to TGF-βRI. Such a reduction in dTAG-SMAD3 phosphorylation could be confused for successful targeted dephosphorylation. To avoid this confounding possibility, we conceptualized a washout-style assay (Figure 2B) based on our observations in Figure S1. With this washout-style assay, we sought to establish maximal dTAG-SMAD3 phosphorylation first by stimulating cells with TGF-β for 1 h, before either lysing cells to capture this maximal dTAG-SMAD3 phosphorylation (termed 0 h after washout) or washing out TGF-β stimulation and treating cells with either BDPIC or DMSO control for 2 h before lysis (termed 2 h after washout). In the 2 h after washout samples, the rate of dTAG-SMAD3 dephosphorylation could be compared between BDPIC and DMSO treatments, with successful targeted dephosphorylation of dTAG-SMAD3 by BDPIC-mediated recruitment of a bromoTAG-phosphatase expected to accelerate the rate of dTAG-SMAD3 dephosphorylation compared to DMSO-treated controls. We chose to compare the rates of dTAG-SMAD3 dephosphorylation between BDPIC- and DMSO-treated cells at 2 h after TGF-β washout since we reasoned that the ∼60% reduction in phospho-dTAG-SMAD3 levels observed at this time point (Figures S1C and S1D) would, compared to longer time durations, enable any accelerated dephosphorylation to be detectable. To explore a range of phosphatase modalities and substrate specificities, we selected four bromoTAG-phosphatases, including PPP1CA, PPP2CA, PPM1A, and PPM1H. PPP1CA and PPP2CA operate as holoenzyme complexes, involving a regulatory subunit (and a scaffolding subunit for PPP2CA).^37^^,^^38^^,^^39^ In contrast, the catalytic activity of PPM1A and PPM1H is not dependent on holoenzyme complex formation, with monomeric or dimeric functions proposed for these phosphatases.^40^^,^^41^ Previously, PPM1A has been reported as a physiological phosphatase of SMAD3.^42^ PP2A has also been reported to dephosphorylate SMAD3 under hypoxic conditions.^43^ Compared to DMSO treatment 2 h after TGF-β washout, both 100 nM and 500 nM BDPIC caused a marked acceleration in the dephosphorylation of dTAG-SMAD3 in cells stably co-expressing bromoTAG-PPM1A or bromoTAG-PPM1H (Figure 2C, quantified in Figure S2A). Cells stably co-expressing bromoTAG-PPP2CA or bromoTAG-PPP1CA did not exhibit the same substantial acceleration in dTAG-SMAD3 dephosphorylation upon BDPIC treatment (Figure 2C, quantified in Figure S2A). No effect was observed on the phosphorylation of endogenous SMAD3, which lacks the dTAG, with BDPIC treatment in any of the cells, indicating that BDPIC specifically accelerates the dephosphorylation of dTAG-SMAD3.

To confirm that the accelerated dTAG-SMAD3 dephosphorylation was due to recruitment of the phosphatase activity of bromoTAG-phosphatases, no-phosphatase controls as well as previously reported catalytically inactive point mutants were employed: PPM1H^H153D^, PPM1A^D239A^, and PPP2CA^H118Q.^^44^^,^^45^^,^^46^^,^^47^^,^^48^^,^^49^ In the absence of any bromoTAG-phosphatase, BDPIC did not yield a marked acceleration in dTAG-SMAD3 dephosphorylation compared to DMSO treatment in U2OS cells stably expressing only dTAG-SMAD3 (Figures 2D–2F, quantified in Figures S2B–S2D). For bromoTAG-PPM1H, the catalytically inactive mutant, bromoTAG-PPM1H^H153D^ was unable to elicit the same acceleration in dTAG-SMAD3 dephosphorylation upon BDPIC treatment, in comparison to DMSO treatment, confirming that recruitment of the phosphatase catalytic activity of bromoTAG-PPM1H is required for BDPIC-induced dephosphorylation (Figure 2D, quantified in Figure S2B). For bromoTAG-PPM1A, however, a partial acceleration in the rate of dTAG-SMAD3 dephosphorylation was still observed following BDPIC treatment of cells co-expressing the catalytically inactive bromoTAG-PPM1A^D239A^ mutant (Figure 2E, quantified in Figure S2C). Our observations imply that either the PPM1A^D239A^ mutant is not fully catalytically inactive, or that it recruits phosphatase activity, for example through dimerization with endogenous PPM1A, to dephosphorylate dTAG-SMAD3. For bromoTAG-PPP2CA, minimal difference in dTAG-SMAD3 phosphorylation was observed upon BDPIC treatment over DMSO controls in cells expressing dTAG-SMAD3 alone or those co-expressing dTAG-SMAD3 with either bromoTAG-PPP2CA or the catalytically inactive bromoTAG-PPP2CA^H118Q^ mutant (Figure 2F, quantified in Figure S2D). Given these findings, we decided not to pursue bromoTAG-PPM1A or bromoTAG-PPP2CA further. Phospho-dTAG-SMAD3 and phospho-endogenous SMAD3 levels remained largely similar across DMSO-treated cells expressing either dTAG-SMAD3 alone or in combination with an active bromoTAG-phosphatase. This suggests that phosphorylation of both dTAG-SMAD3 and endogenous SMAD3 was not affected simply by stable expression of any of the bromoTAG-phosphatases employed, despite some having been reported as physiological SMAD3 phosphatases.^42^^,^^43^ As our observations clearly demonstrated that bromoTAG-PPM1H catalytic activity can be redirected to target dTAG-SMAD3 dephosphorylation, we sought to characterize this further.

### Development of HDPIC to induce proximity between Halo-SMAD3 and dTAG-phosphatases

Prior to the development of BDPIC, we generated another bifunctional molecule for proof-of-concept exploration of chemically induced targeted protein dephosphorylation, comprising the established ligands binding to HaloTag^50^ and dTAG^30^^,^^35^ connected by a small PEG linker (Figures S3A and S3B). We named this compound HDPIC (HaloTag-dTAG proximity-inducing chimera) and observed no obvious cytotoxicity when U2OS and A549 cells were treated with a range of concentrations (0–16 μM) of HDPIC for 24 h, in contrast to positive control MG132 treatment, which caused cell death (Figure S3C). We used a previously established method to probe engagement of HDPIC with a Halo-protein in cells, whereby the small (∼35 kDa) artificial Halo-tagged protein FLAG-NLS(nuclear localization signal)-Halo-HiBiT undergoes a small molecular weight shift upon covalent binding of bifunctional molecules containing the Halo-ligand.^34^ Using this assay in U2OS cells stably expressing FLAG-NLS-Halo-HiBiT, 1 μM HDPIC induced a robust mobility shift in FLAG-NLS-Halo-HiBiT (Figure S3D), indicating both cell-permeability of HDPIC and HaloTag engagement. To assess engagement of HDPIC with a dTAG-protein in cells, we showed that HDPIC could partially rescue the degradation of dTAG-PPP2CA mediated by the dTAG-13 PROTAC^30^ through competitive binding in HEK293 ^*dTAG/dTAG*^*PPP2CA* knock-in cells^51^ (Figure S3E). This indicates that HDPIC engages dTAG in cells. Given that HDPIC demonstrated interaction with both Halo-protein and dTAG-protein *in cellulo*, we sought to explore whether HDPIC could be employed to target dephosphorylation of Halo-SMAD3 by inducing proximity to a dTAG-phosphatase. To this end, we tested the effect of HDPIC on the rate of dephosphorylation of Halo-SMAD3 in U2OS cells stably expressing Halo-SMAD3 alone or in combination with dTAG-PPM1H (Figure S3F). Using a similar washout-style assay as before (Figures 2B–2F), cells were stimulated with TGF-β for 1 h and following washout, treated with DMSO or HDPIC for 2 h or 4 h prior to lysis. Unexpectedly, in U2OS cells expressing Halo-SMAD3 alone, even in the absence of dTAG-phosphatase 1 μM HDPIC treatment for both 2 h and 4 h accelerated Halo-SMAD3 dephosphorylation in comparison to untreated or DMSO-treated controls (Figure S3F, quantified in Figure S4). No difference was observed between untreated and DMSO-treated cells, confirming that DMSO solvent (in which HDPIC was dissolved) is not responsible for the accelerated dephosphorylation observed in HDPIC-treated cells. Furthermore, no additional increase in the rate of Halo-SMAD3 dephosphorylation was observed upon co-expression of dTAG-PPM1H (Figure S3F, quantified in Figure S4), suggesting that the accelerated dephosphorylation observed is due to HDPIC engagement with Halo-SMAD3. We observed a similar, but less marked, effect in A549 ^*Halo/WT*^*SMAD3* knock-in cells (validated by immunoblotting, PCR and genomic sequencing (Figures S3G–S3I)), where, despite no dTAG-phosphatase expression, HDPIC treatment mildly accelerated Halo-SMAD3 dephosphorylation 4 h after TGF-β washout compared to DMSO-treated controls (Figure S3J). A molecule similar to HDPIC but differing slightly in the linker length and in the attachment position of the linker to the dTAG ligand, termed PhosTAC7 (Figure S3K), was reported to induce proximity between dTAG-PP2A-A subunit and Halo-PDCD4 and Halo-FOXO3a for targeted Halo-protein dephosphorylation.^25^ Like HDPIC, PhosTAC7 treatment accelerated the dephosphorylation of Halo-SMAD3 in comparison to DMSO-treated cells, even in the absence of a dTAG-phosphatase (Figure S3J). Together, these data indicate that the accelerated dephosphorylation observed with HDPIC or PhosTAC7 treatment in comparison to DMSO is a compound-dependent effect without the need for phosphatase recruitment. This could potentially result from chloroalkane binding covalently to Halo-SMAD3, or due to interference of the TGF-β pathway by HDPIC or PhosTAC7 in such a manner that reduces Halo-SMAD3 phosphorylation. It is worth noting that this compound-dependent effect is likely to be target dependent, since similar evidence of dephosphorylation upon chloroalkane compound treatment was not observed for transcription factor EB (TFEB)-Halo in a parallel work.^52^

It should also be noted that the negative control compound that we synthesized, HDPIC-Neg (Figure S5A), in which the HaloTag-binding chloroalkane is substituted for a fluoroalkane, and which yielded no obvious cytotoxicity in U2OS or A549 cells (Figure S5B), displayed evidence of Halo-POI engagement at micromolar concentrations (Figure S5C). Similar to HDPIC, HDPIC-Neg treatment of U2OS FLAG-NLS-Halo-HiBiT cells resulted in the appearance of a higher molecular weight mobility shift in FLAG-NLS-Halo-HiBiT, which is observed upon covalent engagement of chloroalkane with the HaloTag^34^ (Figure S5C). The intensity of the upper band for FLAG-NLS-Halo-HiBiT was weaker in cells treated with HDPIC-Neg than HDPIC, suggesting that HDPIC-Neg still engages FLAG-NLS-Halo-HiBiT, albeit weakly and at higher concentrations. Since exploration with HDPIC or PhosTAC7 did not indicate any signs of targeted dephosphorylation through induced proximity between Halo-SMAD3 and dTAG-phosphatase, we focused our efforts on BDPIC.

### BDPIC at 100 nM efficiently targets dTAG-SMAD3 dephosphorylation through bromoTAG-PPM1H recruitment

**Figure 3:**
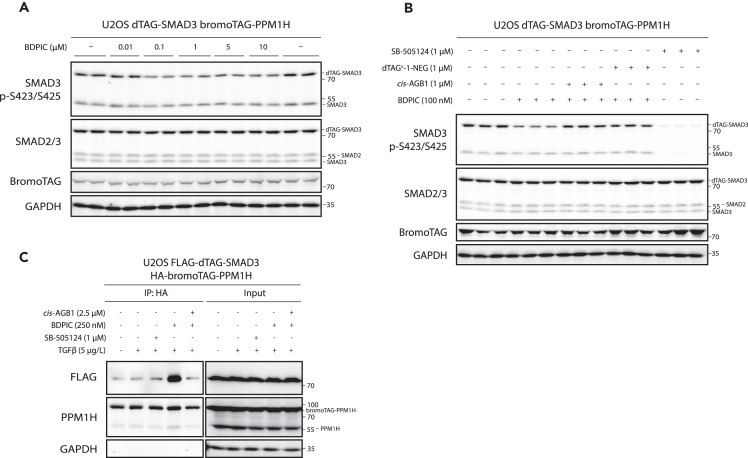
Characterization of BDPIC-mediated targeted dephosphorylation of dTAG-SMAD3 through recruitment of bromoTAG-PPM1H (A) U2OS cells co-expressing dTAG-SMAD3 and bromoTAG-PPM1H were serum-starved overnight before 2 h co-treatment with TGF-β (5 μg/L) and the indicated concentrations of BDPIC or DMSO. Extracts were resolved by SDS-PAGE, transferred to nitrocellulose membrane, and subjected to immunoblotting with the indicated antibodies. Three independent experiments are shown together. (B) U2OS cells co-expressing dTAG-SMAD3 and bromoTAG-PPM1H were serum-starved overnight before 2 h co-treatment of TGF-β (5 μg/L) and the indicated compounds or DMSO. Extracts were processed as in (A). (C) U2OS cells co-expressing FLAG-dTAG-SMAD3 and HA-bromoTAG-PPM1H were serum-starved overnight before 2 h treatment with either control or TGF-β (5 μg/L) in addition to DMSO, SB-505125 (1 μM), BDPIC (250 nM) or a combination of BDPIC (250 nM) and *cis*-AGB1 (2500 nM). Cells were then lysed and extracts or anti-HA-immunoprecipitates (IP) were processed for immunoblotting as in (A). Data are representative of *n* = 3 independent experiments.

To further characterize the targeted dephosphorylation of dTAG-SMAD3 through BDPIC-mediated recruitment of bromoTAG-PPM1H, a dose response experiment was conducted in U2OS cells stably co-expressing dTAG-SMAD3 and bromoTAG-PPM1H. With 2 h treatment, BDPIC concentrations of 0.1–10 μM reduced the phosphorylation of dTAG-SMAD3 by around 50% in comparison to DMSO-treated controls (Figure 3A, quantified in Figure S6). Interestingly, a hook effect^53^ was not observed with any of the tested concentrations. Concentrations of BDPIC higher than 10 μM were not explored due to precipitation when applying to aqueous medium. Given the robust dephosphorylation yielded with 100 nM BDPIC, we continued with this concentration for further exploration of BDPIC-mediated targeted dTAG-SMAD3 dephosphorylation through recruitment of bromoTAG-PPM1H.

### BDPIC-mediated targeted dephosphorylation of dTAG-SMAD3 through recruitment of bromoTAG-PPM1H is rescued by competition with *cis*-AGB1 and dTAG^V^-1-NEG

To confirm that the BDPIC-mediated targeted dephosphorylation of dTAG-SMAD3 observed in Figures 2C and 2D and 3A is reliant on the recruitment of bromoTAG-PPM1H, a competition assay was conducted using *cis-*AGB1, an inactive enantiomer of the AGB1 PROTAC^32^ that shares the same bromoTAG ligand as BDPIC, and dTAG^V^-1-NEG, an inactive enantiomer of the dTAG^V^-1 PROTAC^54^ that shares the same dTAG ligand as BDPIC. U2OS cells stably co-expressing dTAG-SMAD3 and bromoTAG-PPM1H were serum-starved prior to 2 h treatment with TGF-β and either DMSO, TGF-βRI inhibitor SB-505124, BDPIC (100 nM), or BDPIC in combination with either excess *cis*-AGB1 (1 μM) or excess dTAG^V^-1-NEG (1 μM) (Figure 3B). As expected, SB-505124 treatment led to full attenuation of both dTAG-SMAD3 and endogenous SMAD3 tail phosphorylation in comparison to DMSO-treated controls. BDPIC treatment led to a robust reduction in phosphorylation of dTAG-SMAD3 in comparison to DMSO-treated controls, with no effect apparent on endogenous SMAD3 phosphorylation. Co-treatment with BDPIC and excess *cis*-AGB1 almost completely prevented the BDPIC-induced dephosphorylation of dTAG-SMAD3. Similarly, co-treatment with BDPIC and excess dTAG^V^-1-NEG also rescued the BDPIC-induced dephosphorylation of dTAG-SMAD3. The levels of phospho-dTAG-SMAD3 in cells co-treated with BDPIC and *cis-*AGB1 or dTAG^V^-1-NEG were comparable to those seen in cells treated with DMSO (Figure 3B). This confirms that BDPIC-mediated targeted dephosphorylation of dTAG-SMAD3 is reliant on BDPIC simultaneously binding to and recruiting bromoTAG-PPM1H and dTAG-SMAD3.

### BDPIC induces ternary complex formation with bromoTAG-PPM1H and dTAG-SMAD3

To validate that BDPIC could mediate ternary complex formation between bromoTAG-PPM1H and dTAG-SMAD3 that enables targeted dephosphorylation, we tested whether FLAG-dTAG-SMAD3 could be co-precipitated with HA-bromoTAG-PPM1H in the presence of BDPIC (Figure 3C). Extracts from U2OS cells stably co-expressing FLAG-dTAG-SMAD3 and HA-bromoTAG-PPM1H and treated either with DMSO, SB-505124, BDPIC (250 nM), or a combination of BDPIC and excess *cis*-AGB1 (2.5 μM) were subjected to anti-HA-immunoprecipitation (IP). A marked co-precipitation of FLAG-dTAG-SMAD3 was detected in anti-HA IP samples from BDPIC-treated cells but not from those treated with DMSO, SB-505124, or BDPIC+*cis*-AGB1 (Figure 3C), confirming that BDPIC induces an interaction between dTAG-SMAD3 and bromoTAG-PPM1H.

### Targeted dephosphorylation of dTAG-SMAD3 by BDPIC-mediated recruitment of bromoTAG-PPM1H inhibits TGF-β signaling

**Figure 4:**
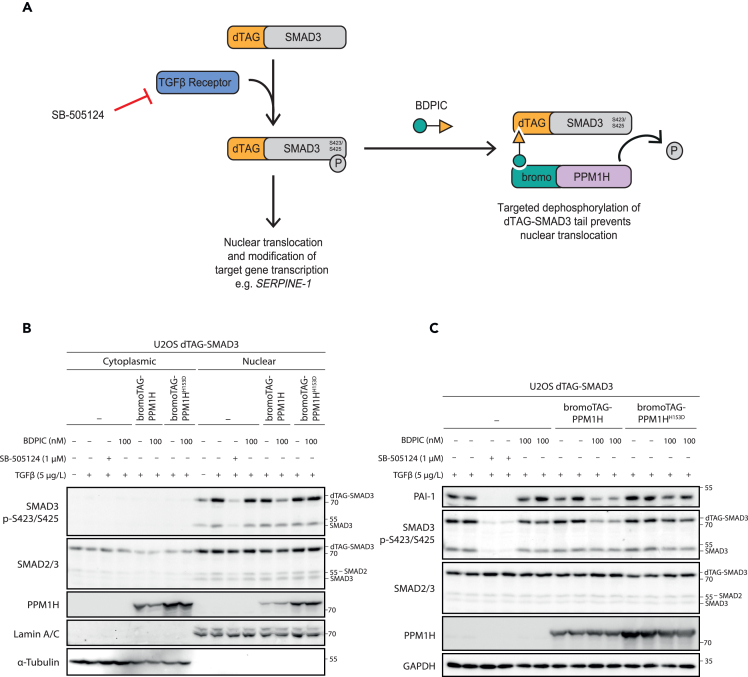
BDPIC-mediated targeted dephosphorylation of dTAG-SMAD3 through bromoTAG-PPM1H recruitment affects the biological function of dTAG-SMAD3 (A) Schematic representation of phospho-dependent function of dTAG-SMAD3 upon TGF-β stimulation and the impact of BDPIC-mediated dTAG-SMAD3 dephosphorylation. (B) dTAG-SMAD3 nuclear translocation was probed by cellular fractionation. U2OS cells were retrovirally transduced to express dTAG-SMAD3 alone or in combination with either bromoTAG-PPM1H or bromoTAG-PPM1H^H153D^. Cells were serum-starved (16 h) prior to 2 h co-treatment with control or TGF-β (5 μg/L), SB-505124 (1 μM) and BDPIC (100 nM) as indicated. Cytoplasmic and nuclear fractions were then isolated before extracts were resolved by SDS-PAGE, transferred to nitrocellulose membrane and subjected to immunoblotting with the indicated antibodies. (C) The impact of dTAG-SMAD3 dephosphorylation on PAI-1 protein levels was investigated by serum-starving U2OS cells expressing dTAG-SMAD3 alone or in combination with bromoTAG-PPM1H or bromoTAG-PPM1H^H153D^, before 6 h treatment with TGF-β (5 μg/L) and DMSO, SB-505124 (1 μM) or BDPIC (100 nM). Cells were lysed and extracts were processed as in (B). Here, duplicate samples are shown together. Data are representative of *n* = 3 independent experiments.

We next explored the impact of targeted dephosphorylation of dTAG-SMAD3 on downstream biology (Figure 4A). Tail phosphorylation of SMAD3 by TGF-βRI triggers a nuclear translocation of SMAD3, enabling regulation of TGF-β target gene transcription.^1^^,^^55^^,^^56^ U2OS cells stably expressing dTAG-SMAD3 alone or in combination with either bromoTAG-PPM1H or bromoTAG-PPM1H^H153D^ mutant were treated for 2 h with control or TGF-β (5 μg/L) along with DMSO, SB-505124 (1 μM), or BDPIC (100 nM) before nuclear and cytoplasmic fractions were collected (Figure 4B). As expected, no phosphorylated dTAG-SMAD3 was evident in any cytoplasmic fractions, regardless of the treatments (Figure 4B). TGF-β stimulation of U2OS cells expressing only dTAG-SMAD3 resulted in substantial accumulation of phospho-dTAG-SMAD3 and endogenous phospho-SMAD3 in the nuclear fraction, whereas additional SB-505124 treatment inhibited this.^4^^,^^5^ Co-treatment of cells expressing only dTAG-SMAD3 with TGF-β and BDPIC yielded no substantial change in nuclear abundance of phospho-dTAG-SMAD3 or endogenous phospho-SMAD3 in comparison to cells treated with TGF-β and DMSO (Figure 4B). Upon co-treatment with TGF-β and DMSO, U2OS cells stably co-expressing dTAG-SMAD3 and bromoTAG-PPM1H displayed similar phospho-dTAG-SMAD3 nuclear abundance as U2OS cells stably expressing only dTAG-SMAD3, suggesting that stable bromoTAG-PPM1H expression does not impede the phosphorylation or nuclear translocation of dTAG-SMAD3 or endogenous SMAD3 (Figure 4B). Excitingly, a distinct reduction in nuclear phospho-dTAG-SMAD3, but not endogenous phospho-SMAD3, was observed in BDPIC-treated U2OS cells co-expressing dTAG-SMAD3 and bromoTAG-PPM1H, suggesting that BDPIC-mediated recruitment of bromoTAG-PPM1H and subsequent dTAG-SMAD3 dephosphorylation reduces the accumulation of phospho-dTAG-SMAD3 in the nucleus (Figure 4B). Since endogenous phospho-SMAD3 nuclear abundance remained unchanged with BDPIC treatment in these cells, this reinforces that BDPIC specifically targets bromoTAG-PPM1H to dephosphorylate dTAG-SMAD3. Furthermore, U2OS cells co-expressing dTAG-SMAD3 and catalytically inactive bromoTAG-PPM1H^H153D^ displayed no reduction in nuclear abundance of phospho-dTAG-SMAD3 upon BDPIC treatment in comparison to cells only expressing dTAG-SMAD3 (Figure 4B). This indicates that the reduction observed in cells co-expressing dTAG-SMAD3 and bromoTAG-PPM1H upon BDPIC treatment is reliant on PPM1H phosphatase activity.

To further explore the impact of targeted dephosphorylation of dTAG-SMAD3, we focused on a well-characterized TGF-β-induced, SMAD3-dependent gene *SERPINE-1*, which encodes PAI-1.^17^ For this, U2OS cells stably expressing dTAG-SMAD3 alone or in combination with bromoTAG-PPM1H or bromoTAG-PPM1H^H153D^ were co-treated with TGF-β and either DMSO, SB-505124, or BDPIC (100 nM) for 6 h (Figure 4C, quantified in Figure S7). TGF-β stimulation of U2OS cells expressing dTAG-SMAD3 alone yielded robust phosphorylation of both dTAG-SMAD3 and endogenous SMAD3 and increased protein levels of PAI-1, all of which were completely attenuated by SB-505124 co-treatment. In TGF-β-stimulated U2OS cells co-expressing dTAG-SMAD3 and bromoTAG-PPM1H, BDPIC treatment induced dephosphorylation of dTAG-SMAD3 and a marked reduction in PAI-1 levels in comparison to DMSO-treated controls (Figure 4C). This suggests that BDPIC-mediated dTAG-SMAD3 dephosphorylation, through bromoTAG-PPM1H recruitment, impedes the transcription of SMAD3-target gene *SERPINE-1*, likely due to the concurrent reduction of nuclear phospho-dTAG-SMAD3 abundance observed in Figure 4B. Although a substantially greater reduction was observed in both dTAG-SMAD3 phosphorylation and PAI-1 protein levels in cells co-expressing dTAG-SMAD3 and bromoTAG-PPM1H upon BDPIC treatment, some reduction was also observed in U2OS cells expressing dTAG-SMAD3 alone and in those also expressing bromoTAG-PPM1H^H153D^ (quantified in Figure S7). We speculate that BDPIC may potentially impact the rate of dTAG-SMAD3 dephosphorylation, which is more apparent with longer BDPIC treatment duration of 6 h compared to 2 h (Figures 4B and 2C–2F). This effect may be due to either the engagement of BDPIC with the dTAG on SMAD3, or an off-target effect of BDPIC on some component(s) of the TGF-β signaling pathway that leads to a slight reduction of SMAD3 phosphorylation. The latter is supported by a slight decrease in the phosphorylation of endogenous SMAD3 that was also observed in BDPIC-treated cells in comparison to DMSO-treated controls. However, given that a substantially greater reduction in both dTAG-SMAD3 phosphorylation and PAI-1 levels was observed in U2OS cells co-expressing dTAG-SMAD3 and bromoTAG-PPM1H upon BDPIC treatment, we conclude that the BDPIC-dependent reduction in dTAG-SMAD3 phosphorylation and PAI-1 levels is due to the phosphatase activity of bromoTAG-PPM1H, which elicits dTAG-SMAD3 dephosphorylation upon BDPIC-induced proximity. Our data suggest that induced proximity between phosphatases and POIs can be employed to mediate targeted protein dephosphorylation and that this change in phospho-status can impact downstream signaling.

### BDPIC can target dephosphorylation of dTAG-SMAD3 through recruitment of bromoTAG-PPM1H at the endogenous level

Intrigued to explore whether BDPIC could mediate targeted dephosphorylation of dTAG-SMAD3 by inducing proximity with bromoTAG-PPM1H at the endogenous level, we generated A549 ^*bromoTAG/bromoTAG*^*PPM1H/*^*dTAG/dTAG*^*SMAD3* knock-in cells using CRISPR-Cas9 genome editing. The knock-in cells were verified by immunoblotting (Figure S8A), PCR (Figure S8B), and DNA sequencing (Figure S8C). Molecular weight shifts of native SMAD3 and PPM1H corresponding to the added dTAG and bromoTAG, respectively, were evident in the knock-in cells relative to the wild-type controls (Figures S8A and S8B). Of note, in the knock-in cells, we also detected a very weak signal for a higher molecular weight species with anti-SMAD3 immunoblotting, although we did not detect any aberrant dTAG-SMAD3 species by DNA sequencing. More importantly, we showed that the knocked-in dTAG-SMAD3 responded to TGF-β stimulation in a similar manner to that expected of endogenous SMAD3 and yielded the expected increase in PAI-1 levels (Figure S8D).

**Figure 5:**
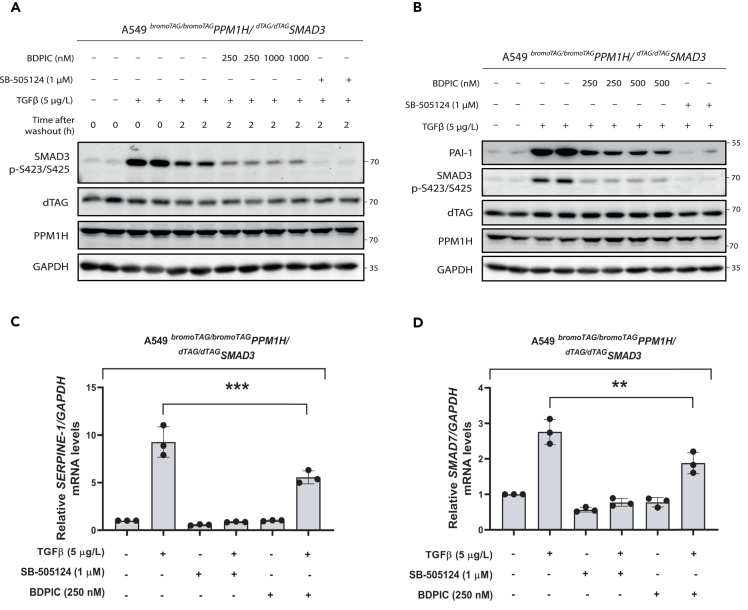
Employing BDPIC to target the dephosphorylation of dTAG-SMAD3 through recruitment of bromoTAG-PPM1H at the endogenous level in A549 ^*bromoTAG/bromoTAG*^*PPM1H/*^*dTAG/dTAG*^*SMAD3* cells (A) BDPIC was employed to mediate targeted dephosphorylation of dTAG-SMAD3 in A549 ^*bromoTAG/bromoTAG*^*PPM1H/*^*dTAG/dTAG*^*SMAD3* knock-in cells (cl. 51). Following serum-starvation (16 h), cells were stimulated with control or TGF-β (5 μg/L) for 1 h. 0 h time points were lysed at this moment. For 2 h time points, TGF-β stimulation was removed by washout and fresh serum-free medium without TGF-β was added to cells, along with DMSO or BDPIC (250 nM or 1,000 nM) for 2 h. Cells were then lysed before extracts were resolved by SDS-PAGE, transferred to nitrocellulose membrane and subjected to immunoblotting with the indicated antibodies. Two biological replicates per treatment condition are shown here. (B) Exploration of BDPIC’s ability to impact production of PAI-1, which is encoded by the SMAD3-dependent gene *SERPINE-1,* by dephosphorylating dTAG-SMAD3 at the endogenous level. A549 ^*bromoTAG/bromoTAG*^*PPM1H/*^*dTAG/dTAG*^*SMAD3* cells were serum-starved (16 h) prior to co-treatment (6 h) with control or TGF-β (5 μg/L) and DMSO, SB-505124 (1 μM), or BDPIC (250 nM or 500 nM) as indicated. Cells were then lysed and extracts were processed as in (A) Two biological replicates per treatment condition are shown here. (C and D) RT-qPCR was performed on serum-starved A549 ^*bromoTAG/bromoTAG*^*PPM1H/*^*dTAG/dTAG*^*SMAD3* cells treated for 6 h with or without TGF-β (5 μg/L) and DMSO, BDPIC (250 nM), or SB-505124 (1 μM) to investigate BDPIC’s impact on the transcription of TGF-β target genes, *SERPINE-1* (C) and *SMAD7* (D) Data are shown as mean ± SD of target gene transcripts normalized to *GAPDH*, relative to control-treated cells (column 1), from *n* = 3 independent experiments. Each experiment included three technical replicates. Statistical analysis involved one-way analysis of variance (ANOVA) with Tukey’s multiple comparisons post-hoc test. Data are representative of *n* = 3 independent experiments (∗∗∗ = p < 0.001 and ∗∗ = p < 0.01).

A similar targeted dephosphorylation assay as before (Figures 2B and 2C) was conducted in A549 ^*bromoTAG/bromoTAG*^*PPM1H/*^*dTAG/dTAG*^*SMAD3* knock-in cells (Figure 5A). In comparison to unstimulated cells, TGF-β-treated cells displayed a robust phosphorylation of dTAG-SMAD3, indicating that incorporation of dTAG has not impeded SMAD3 phosphorylation. Phosphorylation was completely attenuated by co-treatment with inhibitor SB-505124. When dTAG-SMAD3 phosphorylation was monitored 2 h after TGF-β washout, the reduction in dTAG-SMAD3 phosphorylation observed with DMSO control treatment was taken as the natural rate of dephosphorylation of dTAG-SMAD3. In comparison to this, cells treated with 250 nM or 1,000 nM BDPIC displayed a greater decrease in phospho-dTAG-SMAD3 (Figure 5A), suggesting that BDPIC can accelerate dephosphorylation of endogenous dTAG-SMAD3 by recruiting endogenous bromoTAG-PPM1H. 250 nM was chosen for BDPIC treatment after observing a slightly more pronounced dephosphorylation of endogenous dTAG-SMAD3 with this concentration in preliminary optimization than with the 100 nM used in the stable expression model (Figure S8E). Little difference was apparent between cells treated with 250 nM or 1,000 nM BDPIC, suggesting both concentrations to be equally as effective (Figure 5A). To explore whether BDPIC could affect the SMAD3-dependent stimulation of PAI-1 production by targeting the dephosphorylation of dTAG-SMAD3 at the endogenous level, a similar experiment was conducted as before (Figure 4C), involving 6 h treatment of A549 ^*bromoTAG/bromoTAG*^*PPM1H/*^*dTAG/dTAG*^*SMAD3* cells (Figure 5B). In comparison to unstimulated cells, TGF-β-treated cells displayed a substantial increase in PAI-1 levels, again confirming that knock-in of dTAG on SMAD3 has not prevented SMAD3-dependent PAI-1 production. In cells stimulated with TGF-β, co-treatment with 250 nM or 500 nM BDPIC resulted in a marked reduction in the levels of PAI-1 as well as of phospho-dTAG-SMAD3, reinforcing the notion that BDPIC-mediated targeted dephosphorylation can inhibit the phospho-dependent function of dTAG-SMAD3 at the endogenous level. Again, little difference was apparent between different BDPIC treatment concentrations and so the lower concentration of 250 nM was used for further experiments. By RT-qPCR (quantitative reverse-transcription PCR), TGF-β-induced transcription of *SERPINE-1* in A549 ^*bromoTAG/bromoTAG*^*PPM1H/*^*dTAG/dTAG*^*SMAD3* cells was significantly decreased upon BDPIC (250 nM) treatment when compared to DMSO-treated controls (Figure 5C). We also monitored the level of transcription of another SMAD3-responsive gene, *SMAD7*, an inhibitory SMAD protein that acts as a negative regulator of canonical TGF-β signaling,^57^^,^^58^ which showed a similar significant decrease upon BDPIC (250 nM) treatment in comparison to DMSO-treated controls (Figure 5D).

### BDPIC-mediated recruitment of bromoTAG-PPM1H leads to dephosphorylation of additional dTAG-SMAD3 phospho-residues

**Figure 6:**
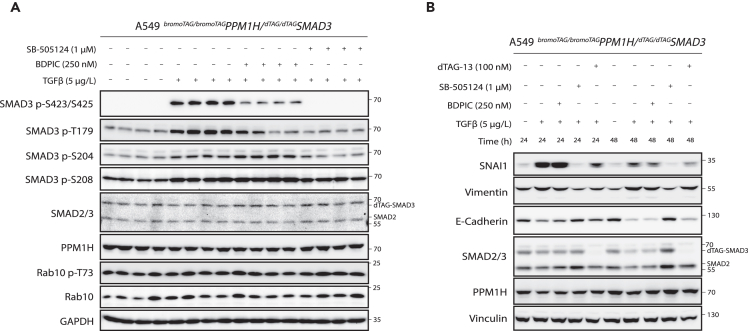
Evaluation of BDPIC-induced targeted dephosphorylation of dTAG-SMAD3 by bromoTAG-PPM1H and its impact on TGF-β-induced epithelial to mesenchymal transition (EMT) (A) A549 ^*bromoTAG/bromoTAG*^*PPM1H/*^*dTAG/dTAG*^*SMAD3* cells were serum-starved (16 h) before treatment with or without TGF-β (5 μg/L) and DMSO, BDPIC (250 nM), or SB-505124 (1 μM) for 6 h prior to lysis. Extracts were resolved by SDS-PAGE, transferred to nitrocellulose membrane and subjected to immunoblotting with the indicated antibodies. Four independent experiments are shown together. (B) A549 ^*bromoTAG/bromoTAG*^*PPM1H/*^*dTAG/dTAG*^*SMAD3* cells were serum-starved (16 h) before treatment with or without TGF-β (5 μg/L) and DMSO, BDPIC (250 nM), SB-505124 (1 μM), or dTAG-13 (100 nM) for 24 h or 48 h prior to lysis, as indicated. Cells were lysed and extracts were processed as in (A) and immunoblotted with the indicated antibodies, which included EMT markers. Data are representative of *n* = 3 independent experiments.

Given that SMAD3 is phosphorylated at multiple residues in addition to the tail motif, we were interested to find out whether BDPIC-mediated recruitment of bromoTAG-PPM1H to dTAG-SMAD3 also results in the dephosphorylation of other phospho-residues on dTAG-SMAD3. Phosphorylation of the SMAD3 linker region at Thr179, Ser204, and Ser208 has been reported to be mediated by kinases such as MAPKs, CDKs, and GSK3β in response to stimuli that activate these kinases.^20^^,^^21^^,^^59^^,^^60^ Phosphorylation of some SMAD3 linker sites can be induced by TGF-β stimulation itself, and this is generally considered essential to negatively regulate SMAD3 transcriptional activity, although some instances of positive regulation have also been reported.^21^^,^^61^^,^^62^ We monitored the phosphorylation of these linker sites following BDPIC treatment (Figure 6A). As before, a robust reduction in TGF-β-induced dTAG-SMAD3 tail phosphorylation was observed in cells treated with BDPIC in comparison to DMSO. Under these conditions, a reduction in dTAG-SMAD3 phosphorylation upon BDPIC treatment was also observed at Thr179 (Figure 6A). This suggests that BDPIC-mediated recruitment of bromoTAG-PPM1H to dTAG-SMAD3 could induce the dephosphorylation of Thr179. However, whether this reduction in Thr179 phosphorylation is a result of targeted dephosphorylation by the recruited bromoTAG-PPM1H, or a consequence of reduced dTAG-SMAD3 tail phosphorylation requires further investigation. In contrast, BDPIC treatment resulted in a slight increase in the phosphorylation of Ser204 and Ser208 in comparison to DMSO-treated cells stimulated with TGF-β (Figure 6A). This could be explained by the BDPIC-recruited bromoTAG-PPM1H potentially impeding access for endogenous linker phosphatases, such as SCP1/2 (small C-terminal domain phosphatase 1/2), to some residues of the linker region of dTAG-SMAD3, such as Ser204 and Ser208, thereby increasing their phosphorylation.^63^^,^^64^ Further work is required to confirm these possibilities.

### Redirecting bromoTAG-PPM1H to dephosphorylate dTAG-SMAD3 by BDPIC has minimal impact on the phosphorylation of *bona fide* PPM1H substrate, Rab10

We sought to test whether redirecting endogenous bromoTAG-PPM1H to dephosphorylate neo-substrate dTAG-SMAD3 could impact the phosphorylation status of its physiological substrates. PPM1H has previously been reported to dephosphorylate some Rab GTPase proteins, including Rab10 at phospho-Thr73 which is phosphorylated by LRRK2 (leucine rich-repeat kinase 2).^41^^,^^49^ We monitored the phosphorylation of Rab10 in A549 ^*bromoTAG/bromoTAG*^*PPM1H/*^*dTAG/dTAG*^*SMAD3* cells treated for 6 h with TGF-β or control and BDPIC (250 nM), SB-505124, or DMSO (Figure 6A). No substantial change in Rab10 phosphorylation at Thr73 was observed in BDPIC-treated cells in comparison to DMSO-treated cells stimulated with TGF-β. This suggests that redirecting bromoTAG-PPM1H by BDPIC to dephosphorylate neo-substrate dTAG-SMAD3 does not appear to prevent dephosphorylation of its physiological substrate Rab10.

### BDPIC-mediated targeted dephosphorylation of dTAG-SMAD3 slightly affects the TGF-β-induced expression of EMT markers

Given the significant reduction of SMAD3 target gene transcription upon BDPIC treatment of A549 ^*bromoTAG/bromoTAG*^*PPM1H/*^*dTAG/dTAG*^*SMAD3* cells, we investigated the downstream physiological consequences of BDPIC-mediated targeted dephosphorylation of dTAG-SMAD3. In certain cancer cells, TGF-β signaling is reported to promote epithelial to mesenchymal transition (EMT) through activation of effectors, such as SNAI1 (also referred to as SNAIL - Snail family transcriptional repressor 1).^65^^,^^66^ We monitored the levels of various epithelial and mesenchymal markers in A549 ^*bromoTAG/bromoTAG*^*PPM1H/*^*dTAG/dTAG*^*SMAD3* cells following 24 h or 48 h treatment with TGF-β in the presence or absence of BDPIC (250 nM), SB-505124, dTAG-13 PROTAC^30^ (100 nM), or DMSO (Figure 6B). In comparison to unstimulated cells, cells stimulated with TGF-β for both 24 h and 48 h displayed increased levels of SNAI1 and mesenchymal marker vimentin, while levels of epithelial marker E-cadherin were decreased. These changes were completely abrogated by treatment with TGF-βRI inhibitor, SB-505124. Consistent with these observations, a marked change from round and cuboid to elongated cell morphology was apparent in cells stimulated for 48 h with TGF-β compared to DMSO- and SB-505124-treated cells (Figure S9). Compared to TGF-β-treated controls, cells treated with TGF-β and BDPIC for 24 h and 48 h displayed slightly lower levels of SNAI1 and vimentin, and higher levels of E-cadherin, suggesting some inhibition of EMT via BDPIC-induced targeted dTAG-SMAD3 dephosphorylation. Although this inhibition was not as robust as that caused by SB-505124, it was to a similar extent as that caused by degradation of dTAG-SMAD3 by dTAG-13, which resulted in a robust and complete degradation of dTAG-SMAD3 but no change to levels of SMAD2. The results suggest that BDPIC-induced dTAG-SMAD3 dephosphorylation inhibits TGF-β-induced SMAD3-dependent EMT.

## Discussion

Here, we describe the development of a bifunctional molecule, BDPIC, that serves to induce proximity between bromoTAG and dTAG in cells. Using nanomolar concentrations of BDPIC, we demonstrate proof-of-concept for targeted dephosphorylation of a dTAG-POI in cells co-expressing a bromoTAG-phosphatase. Specifically, we identify bromoTAG-PPM1H as a promising phosphatase that can be redirected to dephosphorylate dTAG-SMAD3, which, to our knowledge, is not known to be a natural substrate for PPM1H. Importantly, in cell lines stably expressing dTAG-SMAD3, the phosphorylation of endogenous SMAD3 was unaffected, indicating that BDPIC was able to selectively dephosphorylate dTAG-SMAD3. Excitingly, in stable expression cells, BDPIC-induced targeted dephosphorylation of dTAG-SMAD3 through recruitment of bromoTAG-PPM1H inhibited dTAG-SMAD3 nuclear translocation and reduced the protein levels of SMAD3 target gene, *SERPINE-1*. We subsequently confirmed our findings at the endogenous level by using CRISPR-Cas9 technology to generate A549 ^*bromoTAG/bromoTAG*^*PPM1H/*^*dTAG/dTAG*^*SMAD3* knock-in cells, in which we demonstrated that BDPIC can recruit endogenous bromoTAG-PPM1H to target the dephosphorylation of endogenous dTAG-SMAD3. BDPIC treatment resulted in a significant reduction in TGF-β-induced *SERPINE-1* and *SMAD7* transcription in these cells and appeared to impede TGF-β-induced EMT to a similar extent as that caused by a complete degradation of dTAG-SMAD3. We also showed that the phosphorylation of physiological PPM1H substrate, Rab10, was not substantially affected by BDPIC-mediated redirection of bromoTAG-PPM1H to dTAG-SMAD3 in A549 ^*bromoTAG/bromoTAG*^*PPM1H/*^*dTAG/dTAG*^*SMAD3* cells.

In a parallel study, we have demonstrated the versatility of BDPIC by successfully targeting the dephosphorylation of another transcription factor, TFEB-dTAG, through recruitment of a different phosphatase, bromoTAG-PPP2CA, at the endogenous level.^52^ In comparison to other strategies aiming to modulate signaling outcomes, such as the DT-6 PROTAC targeting TGF-β-1^67^ or SB-505124 targeting TGF-βRI,^4^ a phosphatase-redirecting approach, such as BDPIC, offers the ability to achieve substrate-level phospho-control of a POI. In the case of SMAD3, a downstream mediator of TGF-β signaling, a phosphatase-redirecting approach could provide finer tuning of biological outcomes in contrast to inhibitors and antagonists that target upstream components of the TGF-β pathway and impact all ensuing TGF-β signaling.

We also tested alternative bifunctional molecules, HDPIC and PhosTAC7,^25^ that engage HaloTAG and dTAG, but noted a robust decrease in Halo-SMAD3 phosphorylation even in the absence of a dTAG-phosphatase, suggesting an off-target effect of compound binding. For BDPIC, although a minor reduction in dTAG-SMAD3 phosphorylation was observed with long treatment times (>6 h) in the absence of bromoTAG-PPM1H, profound dephosphorylation of dTAG-SMAD3 was evident in the presence of wild type but not catalytically inactive bromoTAG-PPM1H, suggesting targeted dephosphorylation. The small effect of BDPIC on dTAG-SMAD3 phosphorylation is likely due to an effect on some components of TGF-β signaling, as even endogenous SMAD3 phosphorylation was slightly affected upon long treatment time. However, no effect of BDPIC on TFEB phosphorylation in the absence of a bromoTAG-phosphatase was evident.^52^

The choice of phosphatase undoubtedly impacts the outcome of a targeted dephosphorylation approach, be it through the spatial access of the catalytic site to target POI phospho-residue/s once recruited into the ternary complex, subcellular localization, or tissue expression levels. Although involved in almost every cellular process,^68^ bromoTAG-PPP2CA (the catalytic subunit of PP2A) was, perhaps surprisingly, unable to mediate robust dephosphorylation of dTAG-SMAD3 upon BDPIC treatment. Similarly, another broadly acting phosphatase, bromoTAG-PPP1CA (catalytic subunit of phosphatase PP1) did not yield marked dephosphorylation of dTAG-SMAD3. Although possessing catalytic activity, PPP2CA and PPP1CA exist in cells as holoenzyme complexes through association with regulatory subunits (and a scaffolding subunit in the case of PPP2CA).^37^^,^^38^^,^^39^ In contrast, PPM1A or PPM1H are proposed to function as monomeric or dimeric units.^40^^,^^41^ Potentially, these differences in structure of the phosphatases may account for the observed differences in ability to dephosphorylate the phospho-sites on dTAG-SMAD3 that we monitored in this study. It is also possible that these phosphatases may be dephosphorylating other phospho-residues on dTAG-SMAD3 that we did not test.

Here, for our proof-of-concept study, we adopted a tagged approach, whereby we either expressed the tagged proteins stably through retroviral transduction or incorporated the tags at the endogenous locus using CRISPR-Cas9 technology. Tag incorporation is not feasible for every target protein and care must be taken to ensure protein function is unaffected. Indeed, the end goal would be to use a PhosTAC molecule to recruit an endogenous, untagged phosphatase to an endogenous, untagged POI to effectuate POI dephosphorylation and alteration of downstream cell signaling. To this end, a ligand for SMAD3 was reported and, although possessing weak affinity for SMAD3, was incorporated into two bifunctional PROTAC molecules to cause SMAD3 degradation.^69^^,^^70^ This SMAD3 ligand could provide a starting point for a SMAD3-targeting PhosTAC. Excitingly, since protein phosphorylation can both positively and negatively regulate protein function, PhosTACs could afford both loss-of-function and gain-of-function outcomes, depending on which phospho-sites are targeted. BDPIC provides a platform to rapidly test multiple POI-phosphatase combinations to identify effective pairs that can elicit the desired targeted POI dephosphorylation and functional outcomes.

A successful PhosTAC approach offers promise of both a catalytic, sub-stoichiometric mechanism of action (akin to PROTACs) and minimized off-target effects by specifically targeting the substrate POI for dephosphorylation. However, just as there is a focus on increasing the diversity of E3 ligase ligands in the PROTAC field,^71^ expansion of the pool of ligands available for specific, high-affinity recruitment of phosphatases without inhibition of phosphatase activity would greatly benefit the progress of PhosTAC efforts. This would allow hijacking of phosphatases displaying tissue- or disease-specific expression or a specific subcellular distribution and could enable context-specific targeted dephosphorylation. Additional optimization of targeted dephosphorylation could arise from improving compound properties, such as composition and/or length of the linker.

In principle, the bromoTAG and dTAG proximity-inducing chimera molecule we describe here, BDPIC, can be employed in cells to induce spatial proximity between any two accessible proteins of interest. As an example, BDPIC possesses enormous potential for studying other targeted protein post-translational modifications (PTMs) in addition to dephosphorylation, for discovery and drug research. Indeed, in theory, BDPIC could be utilized to recruit a POI to any accessible PTM-modifying enzyme, be that an E3 ligase, DUB (deubiquitinase), kinase, SUMO (small ubiquitin-like modifier) E3, SUMO protease, acetyltransferase, methyltransferase, *O*-GlcNAc transferase, protease or, other. Harnessing endogenous PTM machinery to specifically modulate POI function could provide innovative mechanisms for therapeutics to tackle diseases in which these PTMs are dysregulated.

### Limitations of the study

We synthesized BDPIC based on established binders of dTAG and bromoTAG by introducing a 3-PEG linker. Although the efficacy of our heterobifunctional molecule at nM concentrations and within minutes of administration to cells matches with some of the best published PROTACs, we cannot rule out the possibility that using alternative linkers might lead to more efficacious bromoTAG-dTAG proximity inducers. To evaluate comprehensive off-target effects of BDPIC, unbiased total and phospho-proteomic analyses in parental cells without bromoTAG and dTAG knock-ins would be required and conducting these experiments under TGF-β stimulation conditions could identify any potential off-target of BDPIC to TGF-β signaling components. Unbiased RNA-seq experiments to study the effects of targeted dephosphorylation of SMAD3 by BDPIC would also provide a full scope of the target genes that are affected by SMAD3. The use of BDPIC requires introducing bromoTAG or dTAG on the phospho-POI and the phosphatase by genome editing. As the introduction of tags might affect the function of the proteins, this approach might not be feasible for some target proteins and phosphatases. Formation of ternary complexes between the phosphatase and the target protein is critical for any heterobifunctional molecule to cause targeted dephosphorylation. In this study, PPM1H could be redirected to dephosphorylate phospho-SMAD3 but PPP2CA and PPP1CA could not, whereas in a parallel study we show that PPP2CA can be redirected to efficiently dephosphorylate phospho-TFEB. These observations imply that, in the future, site-specific targeted dephosphorylation could potentially be explored by redirecting different phosphatases to a phospho-target or via altering ternary complexes by modifying the bivalent molecules, for example through altered linkers, in much the same way as PROTACs are modified.

## STAR★Methods

### Key resources table

**Table:** 

REAGENT or RESOURCE	SOURCE	IDENTIFIER
**Antibodies**		

Sheep anti-BRD4 BD2	MRC PPU Reagents & Services	SA599
Sheep anti-dTAG	MRC PPU Reagents & Services	DA179
Rabbit anti-E-cadherin	Cell Signaling Technology	3195S
Rabbit anti-FKBP12	Abcam	ab24373
Mouse anti-FLAG M2 peroxidase	Sigma	A8592-.2MG
Rabbit anti-GAPDH	Proteintech	10494-1-AP
Mouse anti-GAPDH-HRP	Proteintech	HRP-60004
Rat anti-HA-HRP	Roche	11867423001
Mouse anti-HaloTag	Promega	G9211
Rabbit anti-LaminA/C	Cell Signaling Technology	2032S
Rabbit anti-PAI-1	Abcam	ab66705
Sheep anti-PPM1H	MRC PPU Reagents & Services	DA064
Mouse anti-Rab10	nanoTools	0680-100/Rab10-605B11
Rabbit anti-Rab10 p-T73	Abcam	ab230261
Rabbit anti-SMAD2/3	Cell Signaling Technology	8685S
Rabbit anti-SMAD3	Cell Signaling Technology	9523S
Anti-SMAD3 p-T179/SMAD2 p-T220 and anti-SMAD3 p-S204	Kind gift from Dr. Fang Liu (Rutgers University)	N/A
Rabbit anti-SMAD3 p-S208	Antibodies.com	A94166
Rabbit anti-SMAD3 p-S423/S425	Rockland	600-401-919
Rabbit anti-SNAIL	Cell Signalling Technology	3879S
Rat anti-tubulin	Invitrogen	MA1-80189
Rabbit monoclonal Vimentin	Cell Signaling Technology	5741S
Rabbit anti-Vinculin	Abcam	ab129002

**Chemicals, peptides, and recombinant proteins**		

BDPIC	Synthesized by Natalia Shpiro as described in this manuscript	N/A
*cis-*AGB1	Synthesized by Natalia Shpiro as described in this manuscript	N/A
cOmplete EDTA-free protease inhibitor cocktail	Roche	11873580001
Dimethylsulphoxide	Sigma-Aldrich	D8418
dTAG-13	MRC PPU Reagents and Services	N/A
dTAG^V^-1-NEG^54^	Synthesized by Natalia Shpiro as described in this manuscript	N/A
HDPIC and HDPIC-Neg	Synthesized by Natalia Shpiro as described in this manuscript	N/A
MG132	Merck	474790-1MG
MLN4924	MRC PPU Reagents and Services	N/A
PEI MAX – Transfection Grade Linear PEI Hydrochloride MW 40,000	Polysciences	24765
PhosTAC7	Synthesized by Natalia Shpiro as described in this manuscript	N/A
Polybrene (Hexadimethrine bromide)	Sigma-Aldrich	107689
Recombinant TGF-β1	Peprotech	100-21
SB-505124	Sigma	S4696-5MG

**Critical commercial assays**		

CellTox Green Assay	Promega	G8742

**Deposited data**		

Data obtained in this study	This paper	Mendeley Data: https://data.mendeley.com/datasets/tncwwpjbc9/1

**Experimental models: cell lines**		

Human: A549	ATCC	CVCL_0023
Human: HEK293-FT	Invitrogen	R70007
Human: U2OS	ATCC	HTB-96

**Recombinant DNA**		

pBABED FLAG-bromoTAG(L387A)-PPP2CA	MRC PPU Reagents and Services	DU78036
pBABED FLAG-bromoTAG(L387A)-PPP2CA(H118Q)	MRC PPU Reagents and Services	DU78037
pBABED 3FLAG-dTAG-SMAD3	MRC PPU Reagents and Services	DU71496
pBABED FLAG-NLS-Halo-HiBiT	MRC PPU Reagents and Services	DU61387
pBABED 3HA-bromoTAG(L387A)-PPM1A	MRC PPU Reagents and Services	DU79008
pBABED 3HA-bromoTAG(L387A)-PPM1A(D239A)	MRC PPU Reagents and Services	DU77740
pBABED 3HA-bromoTAG(L387A)-PPM1H (hygromycin)	MRC PPU Reagents and Services	DU71835
pBABED 3HA-bromoTAG(L387A)-PPM1H (puromycin)	MRC PPU Reagents and Services	DU78038
pBABED 3HA-bromoTAG(L387A)-PPM1H(H153D)	MRC PPU Reagents and Services	DU77739
pBABED 3HA-bromoTAG(L387A)-PPP1CA	MRC PPU Reagents and Services	DU79009
pBABED 3HA-dTAG-SMAD3	MRC PPU Reagents and Services	DU71724
pBABED HA-dTAG-PPM1H	MRC PPU Reagents and Services	DU77742
pBABED Halo-SMAD3	MRC PPU Reagents and Services	DU77738
pCMV5-Gag/Pol	Cell Biolabs	RV-111
pCMV5-VSV-G	Cell Biolabs	RV-110

**Software and algorithms**		

GraphPad Prism v8	GraphPad	https://www.graphpad.com/scientific-software/prism/

### Resource availability

#### Lead contact

Further information and requests for resources and reagents should be directed to and will be fulfilled by the Lead Contact, Gopal Sapkota (g.sapkota@dundee.ac.uk).

#### Materials availability

All constructs used in this study are available to request from the MRC PPU Reagents & Services webpage (http://mrcppureagents.dundee.ac.uk) and the unique identifier (DU) numbers provide direct links to the cloning strategies and sequence details. All constructs were sequence-verified by the DNA Sequencing Service, University of Dundee (http://www.dnaseq.co.uk).

#### Data and code availability

The datasets generated during this study are available at Mendeley Data (https://data.mendeley.com/datasets/tncwwpjbc9/1). Any additional information required to reanalyze the data reported in this paper is available from the lead contact upon request. This paper does not report original code.

### Experimental models and study participant details

#### Cell lines

All procedures were carried out under aseptic conditions meeting biological safety requirements. A549 cells (ATCC, Cat# CVCL_0023) are human lung adenocarcinoma cells derived from a 58-year-old male, HEK293-FT cells (Invitrogen, Cat# R70007) are a clonal isolate of HEK293 cells transformed with the SV40 large T antigen and U2OS cells (ATCC, Cat# HTB-96) are human epithelial bone osteosarcoma cells derived from a 15-year-old female. For growth, A549, HEK293-FT and U2OS cells were maintained in DMEM (Life Technologies) containing 10% (v/v) fetal bovine serum (FBS, Thermo Fisher Scientific), 2 mM L-glutamine (Lonza), 100 U/mL penicillin (Lonza) and 0.1 mg/mL streptomycin (Lonza). Cells were grown at 37°C with 5% CO_2_ in a water-saturated incubator. For passaging, cells were incubated with trypsin/EDTA at 37°C to detach cells.

### Method details

#### Plasmids

For production of retroviral vectors, the following were cloned into pBABED plasmids: FLAG-bromoTAG(L387A)-PPP2CA (DU78036), FLAG-bromoTAG(L387A)-PPP2CA(H118Q) (DU78037), 3FLAG-dTAG-SMAD3 (DU71496), FLAG-NLS-Halo-HiBiT (DU61387), 3HA-bromoTAG(L387A)-PPM1A (DU79008), 3HA-bromoTAG(L387A)-PPM1A(D239A) (DU77740), 3HA-bromoTAG(L387A)-PPM1H (hygromycin) (DU71835), 3HA-bromoTAG(L387A)-PPM1H (puromycin) (DU78038), 3HA-bromoTAG(L387A)-PPM1H(H153D) (DU77739), 3HA-bromoTAG(L387A)-PPP1CA (DU79009), 3HA-dTAG-SMAD3 (DU71724), HA-dTAG-PPM1H (DU77742), Halo-SMAD3 (DU77738). All constructs were sequence-verified by the DNA Sequencing Service, University of Dundee (http://www.dnaseq.co.uk). These constructs are available to request from the MRC PPU Reagents and Services webpage (http://mrcppureagents.dundee.ac.uk) and the unique identifier (DU) numbers provide direct links to the cloning strategies and sequence details.

#### Retroviral generation of stable cell lines

Retroviral pBABED-puromycin or -hygromycin vectors encoding the desired construct (6 μg) were co-transfected with pCMV5-gag-pol (3.2 μg) and pCMV5-VSV-G (2.8 μg) (Cell Biolabs) into a 10 cm diameter dish of ∼70% confluent HEK293-FT cells. Plasmids were added to 1 mL Opti-MEM medium in addition to 24 μL of 1 mg/mL PEI before gentle mixing and incubation at room temperature for 20 min. The transfection mix was then added dropwise to HEK293-FT cells. 16 h post-transfection, fresh medium was added to the cells. After 24 h, the retroviral medium was collected and passed through 0.45 μm sterile syringe filters. Target cells (∼60% confluent) were transduced with the optimized titer of the retroviral medium diluted in fresh medium (typically 1:1) containing 8 μg/mL polybrene (Sigma-Aldrich) for 24 h. Then the cells were placed in fresh medium containing the appropriate concentration of antibiotic to select cells which had integrated the construct with a control non-transduced plate put under selection in parallel. A pool of transduced cells was utilized for subsequent experiments following complete death of the control plate.

#### Generation of cell lines using CRISPR/Cas9 technology

The CRISPR/Cas9 genome editing system^72^^,^^73^ was used to generate HEK293 homozygous ^*dTAG/dTAG*^*PPP2CA* cells, A549 heterozygous ^*Halo/WT*^*SMAD3* cells and A549 ^*bromoTAG/bromoTAG*^*PPM1H/*^*dTAG/dTAG*^*SMAD3* homozygous cells.

The generation of HEK293 ^*dTAG/dTAG*^*PPP2CA* cells was described previously.^51^ For A549 ^*Halo/WT*^*SMAD3* cells, a pair of gRNAs targeting SMAD3 exon 1 was transfected (DU52711 and DU52710, pX335 sgRNA1 Cas9n and pBABED puromycin U6 sgRNA2) (1 μg each) alongside donor (3 μg) (DU69866 pMA SMAD3 Nter mCherry IRES2 HaloTag) and PEI. For A549 ^*bromoTAG/bromoTAG*^*PPM1H* cells, R. Fasimoye transfected a pair of gRNAs targeting PPM1H exon 1 (1 μg each) (DU64667 and DU64673 pX335 sgRNA1 Cas9n and pBABED puromycin U6 sgRNA2) alongside donor (3 μg) (DU64751 pMK-RQ PPM1H Nter GFP IRES2 BRD4 333-460 L387A donor). For A549 ^*bromoTAG/bromoTAG*^*PPM1H/*^*dTAG/dTAG*^*SMAD3* cells, a pair of gRNAs targeting SMAD3 exon 1 was transfected (1 μg each) (DU52710 and DU52711 pX335 sgRNA1 Cas9n and pBABED puromycin U6 sgRNA2) alongside donor (3 μg) (DU74453 pMA SMAD3 Nter mCherry IRES2 FKBP12^F36V^) and PEI. 16 h post-transfection, selection with 1 μg/mL puromycin (Sigma-Aldrich) was carried out for HEK293 cells, or 3.5 μg/mL for A549 cells, and continued for 48 h. The transfection process was repeated (without a further round of selection). Cells were sorted by flow cytometry and single cells were plated in individual wells of 96-well plates. Viable clones were expanded, and integration of the knock-in at the target locus was verified by Western blotting, polymerase chain reaction (PCR) and genomic sequencing.

#### Treatment of cells with stimuli/compounds

The following reagents were added to the cell medium at concentrations and times indicated in the figures and figure legends: Recombinant TGF-β1 (Peprotech, Cat# 100-21) (reconstituted in 4mM HCl/1 μg/mL BSA), SB-505124 (Sigma, Cat# S4696-5MG), BDPIC (synthesized by Natalia Shpiro), HDPIC (synthesized by Natalia Shpiro), HDPIC-Neg (synthesized by Natalia Shpiro), PhosTAC7^25^ (synthesized by Natalia Shpiro), *cis-*AGB1^32^ (synthesized by Natalia Shpiro), dTAG^V^-1-NEG^54^ (synthesized by Natalia Shpiro), dTAG-13^30^ (MRC PPU Reagents & Services). For serum-starvation, cells were washed with phosphate-buffered saline (PBS) before incubation in serum-free medium for 16 h. Unless stated otherwise in the figure legends, an equivalent volume of 4mM HCl/1 μg/mL BSA was used as a negative control for recombinant TGF-β1 treatment and an equivalent volume of DMSO was used as a negative control for compound treatments.

#### Reverse transcription quantitative PCR (RT-qPCR)

The Qiagen RNeasy kit was used to isolate RNA from cells. cDNA was produced using 1 μg of isolated RNA using the iScript cDNA kit (Bio-Rad) as per the manufacturer’s instructions.

qPCR reactions were performed in triplicate, in 10 μL final volumes for 384-well plates. Each well contained 5 μL of PowerUp SYBR Green mastermix, 1 μL of a 20 μM mixture of both Forward and Reverse primers and 2 μL of cDNA. qPCR was performed with a CFX384 real-time qPCR machine (Bio-Rad). Primers used include (5′-3′): GAPDH Fw (TGCACCACCAACTGCTTAGC), GAPDH Rev (GGCATGGACTGTGGTCATGAG), PAI-1 Fw (AGCTCCTTGTACAGATGCCG), PAI-1 Rev (ACAACAGGAGGAGAAACCCA), SMAD7 Fw (CTGTGCAAAGTGTTCAGGTG) and SMAD7 Rev (TTGAGAAAATCCATCGGGTA).

Data was normalized to the mean of the housekeeping gene (GAPDH) and analyzed using the 2^−ΔΔCt^ method for comparing relative gene expression.^74^ Data analysis and plot generation were carried out using Microsoft Excel and GraphPad Prism software.

#### Cell lysis and immunoprecipitation

Cells were harvested by washing twice with PBS and scraped into ice-cold lysis buffer (50 mM Tris-HCl pH 7.5, 0.27 M sucrose, 150 mM NaCl, 1 mM EGTA, 1 mM EDTA, 1 mM sodium orthovanadate, 10 mM sodium β-glycerophosphate, 50 mM sodium fluoride, 5 mM sodium pyrophosphate and 1% NP-40) supplemented with 1x complete EDTA-free protease inhibitor cocktail (Roche). After incubation for 10 min on ice, lysates were clarified by centrifugation at 17,000 G for 20 min at 4°C. Protein concentration was determined according to the Bradford assay to enable normalization between samples.

For immunoprecipitation (IP), cells were lysed as above and a 1 mL solution containing a minimum of 1 mg protein was then subjected to immunoprecipitation using 20 μL of a 50/50 (v/v) slurry made using HA-frankenbody resin slurry (MRC Reagents and Services) with lysis buffer supplemented with protease inhibitor cocktail. 40 μL was removed for input samples and added to 8 μL 6X SDS sample buffer. IP samples were then incubated for 2 h with rotation at 4°C. Flow-through samples were then removed (40 μL). IP samples were eluted in 40 μL 2X SDS sample buffer and 20 μL of IP and input samples was subjected to immunoblotting. IP:input ratio is ∼25:1.

#### Cytoplasmic/nuclear fractionation

Cells were washed twice with PBS, scraped and pelleted by centrifugation at 4°C (2 min at 1200 rpm). For extraction of the cytoplasmic fraction, cells were resuspended in cytoplasmic lysis buffer (20 mM Tris–HCL (pH 7.5), 0.1 mM EDTA, 2 mM MgCl2, 1% NP40, 50 nM β-glycerophosphate, and 1x cOmplete EDTA-free protease inhibitor cocktail (Roche)). Samples were incubated at room temperature for 2 min and then on ice for 10 min, before centrifugation at 4°C (3000 rpm, 5 min).The supernatant was collected as the cytoplasmic fraction and the pellet was then washed with wash buffer (20 mM Tris–HCL (pH 7.5), 0.1 mM EDTA, 2 mM MgCl2, 50 nM β-glycerophosphate, and 1x complete EDTA-free protease inhibitor cocktail (Roche)) three times. The residual pellet was resuspended in nuclear lysis buffer (20 mM HEPES, 0.4 M NaCl, 25% glycerol, 1 mM EDTA, 0.5 mM NaF, 0.5 mM Na_3_VO_4_, 0.5 mM DTT, and 1x complete EDTA-free protease inhibitor cocktail (Roche)) and the pellet was disrupted by three quick freeze/thaw cycles. Lysates were subsequently incubated on ice for 30 min with regular vortexing, followed by clarification at 17,000 G for 20 min at 4°C with the supernatant then collected as the nuclear fraction.

#### SDS-PAGE and western blotting

Cell lysates containing equal amounts of protein (15–20 μg) were resolved by SDS-PAGE and transferred onto nitrocellulose membrane. Membrane was blocked in 5% (w/v) non-fat milk (Marvel) in TBS-T (50 mM Tris–HCl pH 7.5, 150 mM NaCl, 0.2% Tween 20) and incubated overnight at 4°C in 5% (w/v) BSA/TBS-T or 5% (w/v) milk/TBS-T with the appropriate primary antibodies. Primary antibodies used at indicated dilutions include: anti-BromoTAG (anti-BRD4 BD2) (SA599, MRC PPU Reagents & Services, 1 μg/mL), anti-dTAG (DA179, MRC PPU Reagents and Services, 1 μg/mL), anti-E-cadherin (3195S, CST, 1:1000), anti-FKBP12 (ab24373, Abcam, 1:1000), anti-FLAG M2-Peroxidase (A8592-.2MG, Sigma, 1:500), anti-GAPDH (10494-1-AP Proteintech, 1:5000), anti-GAPDH-HRP (HRP-60004, Proteintech, 1:30000), anti-HA-HRP (11867423001, Roche, 1:1000), anti-HaloTag (G9211, Promega, 1:1000), anti-LaminA/C (2032S, CST, 1:1000), anti-PAI-1 (ab66705, Abcam, 1:1000), anti-PPM1H (DA064, MRC PPU Reagents and Services, 1 μg/mL), anti-Rab10 (nanoTools, 0680–100/Rab10-605B11, 1:1000), anti-Rab10 p-T73 (ab230261, Abcam, 1:1000), anti-SMAD2/3 (8685S, CST, 1:1000), anti-SMAD3 (9523S, CST, 1:1000), anti-SMAD3 p-S208 (A94166, Antibodies.com, 1:500), anti-SMAD3 p-S423/S425 (600-401-919, Rockland, 1:1000), anti-SNAIL (3879S, CST, 1:1000), anti-α-tubulin (MA1-80189, Invitrogen, 1:5000), anti-vimentin (5741S, CST, 1:1000), anti-vinculin (ab129002, Abcam, 1:10000). Anti-SMAD3 p-T179/anti-SMAD2 p-T220 and p-S204 antibodies were a kind gift from Dr. Fang Liu (Rutgers University) and were used at 0.15 μg/mL and 0.5 μg/mL, respectively.^60^^,^^75^^,^^76^

Membrane was subsequently washed with TBS-T and incubated with HRP (horseradish peroxidase)- or IRDye800-conjugated secondary antibody for 1 h at room temperature. HRP-coupled secondary antibodies used at indicated dilutions include: goat anti-rabbit-IgG (7074, CST, 1:5000), rabbit anti-sheep-IgG (31480, Thermo Fisher Scientific, 1:5000), goat anti-rat IgG (62-9520, Thermo Fisher Scientific, 1:5000), goat anti-mouse-IgG (31430, Thermo Fisher Scientific, 1:5000). IRDye800-coupled secondary antibodies used include: IRDye800CW Donkey anti-Rabbit IgG (H + L) (926-32213, Licor, 1:5000). After further washing, signal detection was performed using ECL (Merck) for HRP-conjugated secondaries and ChemiDoc MP System (Bio-Rad). ImageLab (version 6.0.1) (Bio-Rad) was used to analyze protein bands by densitometry.

#### Cell cytotoxicity assay

CellTox Green Assay (Promega, Cat. #G8742) was used to assess the cytotoxicity of BDPIC and HDPIC in A549 and U2OS cells at the indicated concentrations and treatment durations. The fluorescent signal produced by the CellTox Green dye, upon selective binding to the DNA of cells with impaired membrane integrity, is proportional to cytotoxicity. Fluorescence was measured using ex: 480 nm em: 530 nm by a PHERAstar FS plate reader before subtracting blank measurements (made using wells containing medium only, no cells) and normalizing to DMSO treatment. MG132 treatment (20 μM) or the lysis solution from the kit was included as a positive control at each time point. Data was analyzed using Excel (Microsoft) and GraphPad Prism software (Version 8).

#### Bright-field microscopy

Bright-field microscopy images were taken using the ZOE Fluorescent Cell Imaging System (Bio-Rad). Scale bars in the images represent 100 μm.

### Quantification and statistical analysis

Western blot intensities were measured by densitometry analysis using Image Lab and adjusted relative densities were calculated using Excel (Microsoft). GraphPad Prism software (Version 8) was used to generate graphs, which display the mean ± standard deviation (SD) of 3 independent experiments, unless stated otherwise in the figure legend.

RT-qPCR data was normalized to the mean of the housekeeping gene (GAPDH) and analyzed using the 2^−ΔΔCt^ method for comparing relative gene expression.^74^ Data analysis and plot generation were carried out using Microsoft Excel and GraphPad Prism software and details of the statistical analyses performed can be found in the figure legend.

#### Chemistry methods

The methodology for the synthesis of BDPIC (bromoTAG-dTAG proximity-inducing chimera), HDPIC (HaloTAG-dTAG proximity-inducing chimera), and other molecules used in this study is included in the Supplementary Materials (Methods S1).

## Supplemental information

Document S1. Figures S1–S9 and Method S1
