# Supplementary material for: Targeted dephosphorylation of SMAD3 as an approach to impede TGF-β signaling
Source: iScience. 2024 Jul 5;27(8):110423. doi: 10.1016/j.isci.2024.110423 (PMC11298613; doi:10.1016/j.isci.2024.110423)
Supplement: Document S1. Figures S1–S9 and Method S1 [file mmc1.pdf]

iScience, Volume *27*

Supplemental information

Targeted dephosphorylation of SMAD3

as an approach to impede TGF-β signaling

Abigail Brewer, Jin-Feng Zhao, Rotimi Fasimoye, Natalia Shpiro, Thomas J. Macartney, Nicola T. Wood, Melanie Wightman, Dario R. Alessi, and Gopal P. Sapkota

Targeted dephosphorylation of SMAD3 as an approach to impede TGFβ signaling

Abigail Brewer, Jin-Feng Zhao, Rotimi Fasimoye, Natalia Shpiro, Thomas J. Macartney, Nicola T. Wood, Melanie Wightman, Dario R. Alessi and Gopal P. Sapkota*

Supplementary Information

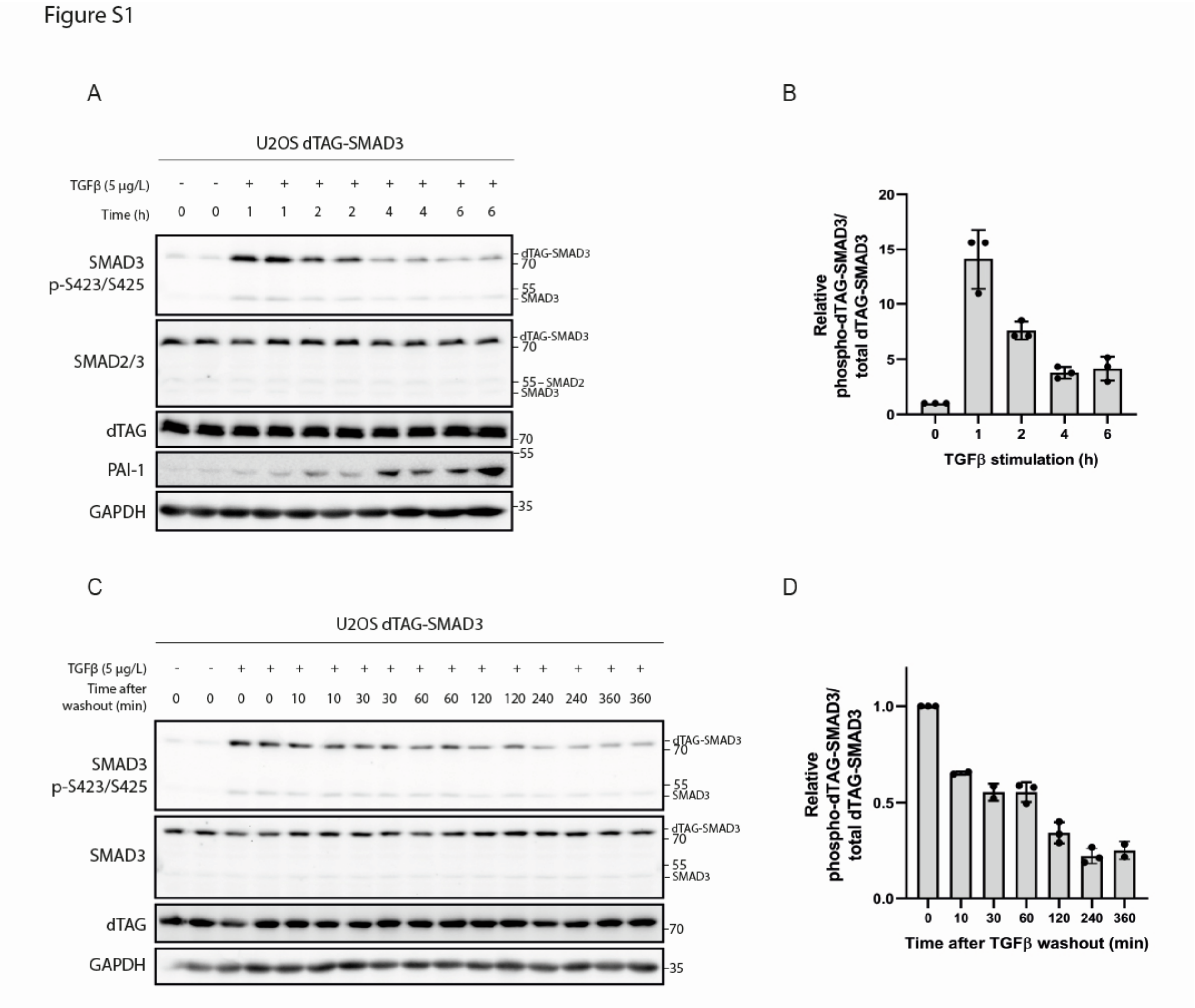

Figure S1: Characterizing dTAG-SMAD3 phosphorylation and dephosphorylation. In relation to Fig. 2.

(**A**) dTAG-SMAD3 phosphorylation was stimulated by treating U2OS cells expressing dTAG-SMAD3 with recombinant TGFβ (5 µg/L) for the indicated durations prior to lysis. Extracts were then resolved by SDS-PAGE, transferred to nitrocellulose membrane and subjected to immunoblotting with the indicated antibodies.

(**B**) Quantification of relative phospho-dTAG-SMAD3/total dTAG-SMAD3 from (**A**). Values are shown as mean ± SD, relative to 0 h treatment.

(**C**) dTAG-SMAD3 dephosphorylation was monitored following 1 h stimulation of U2OS cells expressing dTAG-SMAD3 with TGFβ (5 µg/L), followed by either lysis (0 h after washout) or replacement with fresh serum-free medium and lysis after the indicated time. Unstimulated cells (treated with solution in which TGFβ was reconstituted) were included as a control. Cells were lysed and extracts were processed as in (**A**).

(**D**) Quantification of relative Western blot signal intensities of phospho-dTAG-SMAD3/total dTAG-SMAD3 from (**C**). Values are shown relative to cells treated with TGFβ at 0 h after washout and are displayed as mean ± SD.

Data are representative of n=3 independent experiments (except for 10 min and 30 min time points in (**C**-**D**), which are representative of n=2 independent experiments as these time points were added in later repeats).

Figure S2

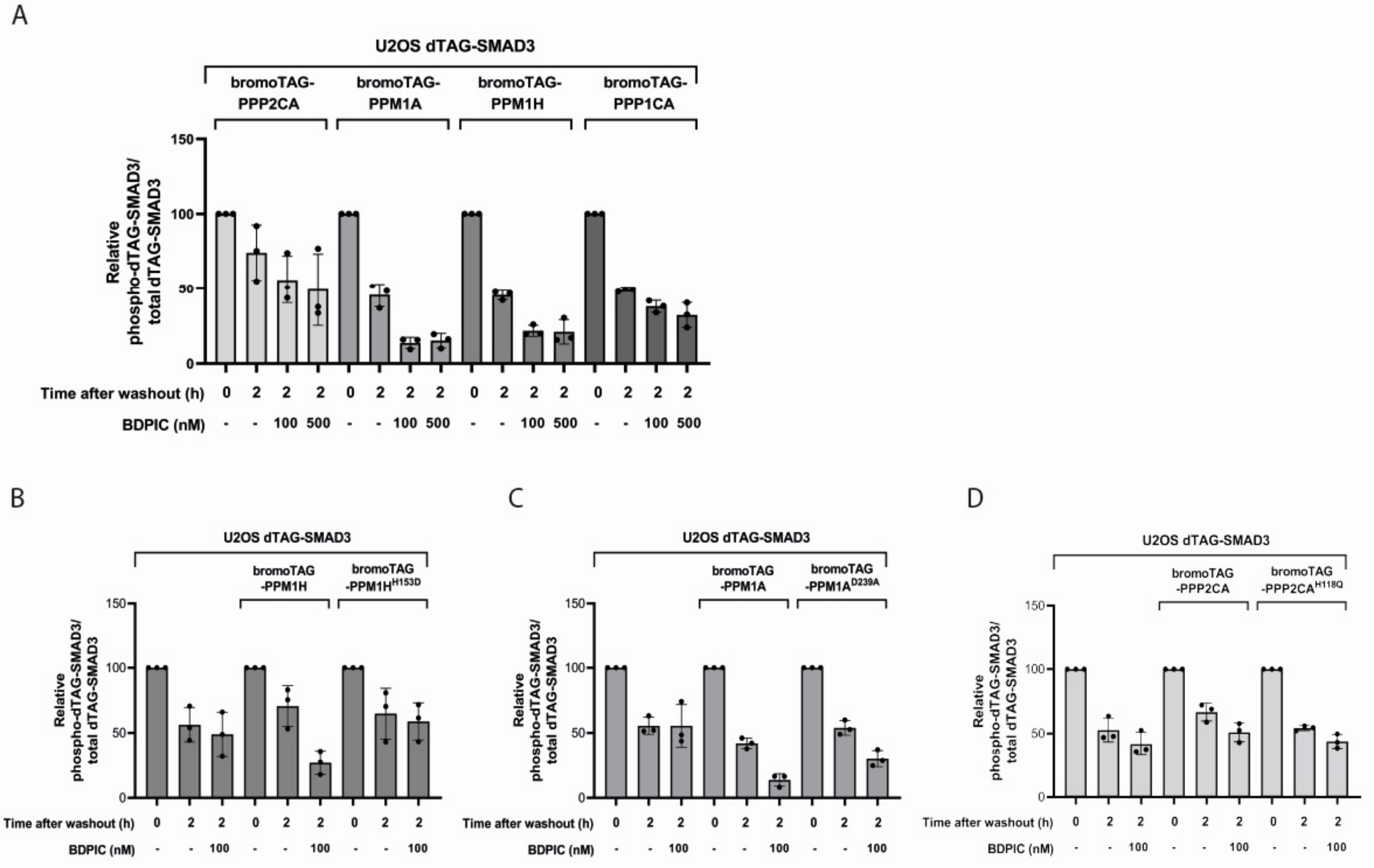

Figure S2: Quantification of relative Western blot signal intensities from biological repeats of Figure 2. In relation to Fig. 2.

(**A**) Quantification of n=3 independent experiments of Fig. 2C, showing mean ± SD phospho-dTAG-SMAD3/total dTAG-SMAD3 levels, relative to the 0 h sample for each cell line.

(**B**) Quantification of n=3 independent experiments of Fig. 2D, showing mean ± SD phospho-dTAG-SMAD3/total dTAG-SMAD3 levels, relative to the 0 h sample for each cell line.

(**C**) Quantification of n=3 independent experiments of Fig. 2E, showing mean ± SD phospho-dTAG-SMAD3/total dTAG-SMAD3 levels, relative to the 0 h sample for each cell line.

(**D**) Quantification of n=3 independent experiments of Fig. 2F, showing mean ± SD phospho-dTAG-SMAD3/total dTAG-SMAD3 levels, relative to the 0 h sample for each cell line.

Figure S3

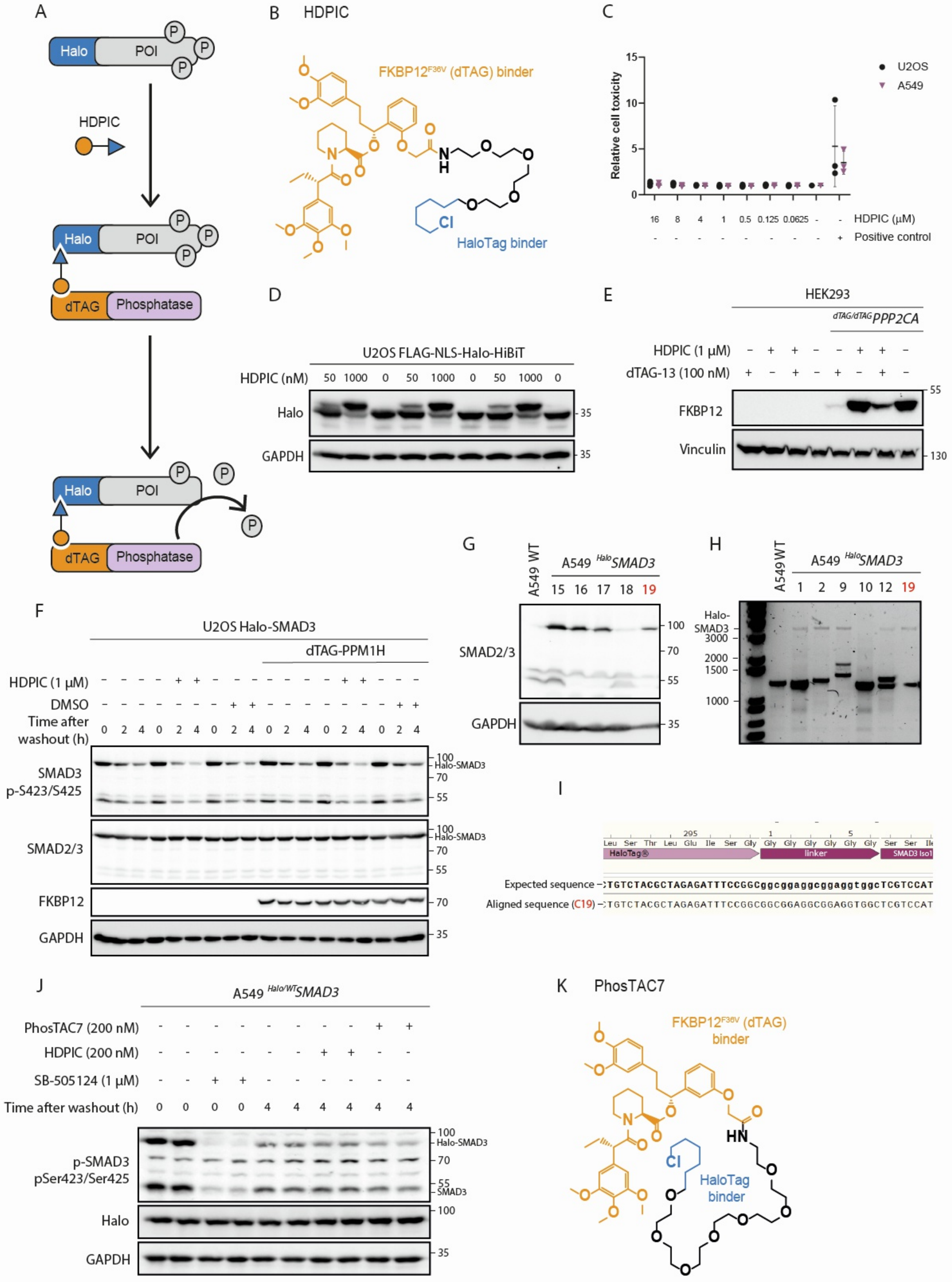

A
Halo
POI
P
HDPIC
dTAG
Phosphatase
B
HDPIC
FKBP12F36V (dTAG) binder
HaloTag binder
C
Relative cell toxicity
U2OS
A549
HDPIC (µM)
16 8 4 1 0.5 0.125 0.0625 - -
Positive control
D
U2OS FLAG-NLS-Halo-HiBiT
HDPIC (nM)
50 1000 0 50 1000 0 50 1000 0
Halo
GAPDH
E
HEK293
dTAG/dTAG PPP2CA
HDPIC (1 µM)
dTAG-13 (100 nM)
FKBP12
Vinculin
F
U2OS Halo-SMAD3
dTAG-PPM1H
HDPIC (1 µM)
DMSO
Time after washout (h)
SMAD3 p-S423/S425
SMAD2/3
FKBP12
GAPDH
Halo-SMAD3
G
A549 WT
A549 HaloSMAD3
15 16 17 18 19
SMAD2/3
GAPDH
H
A549 WT
A549 HaloSMAD3
1 2 9 10 12 19
Halo-SMAD3
3000
2000
1500
1000
I
Leu Ser Thr Leu Glu Ile Ser Gly Gly Gly Gly Gly Gly Gly Ser Ser Ile
HaloTag®
linker
SMAD3 Iso1
Expected sequence
TGTCTACGCTAGAGATTTCCGGCggcggaggcggaggtggcTCGTCCAT
Aligned sequence (C19)
TGTCTACGCTAGAGATTTCCGGCGGCGGAGGCGGAGGTGGCTCGTCCAT
J
A549 Halo/WTSMAD3
PhosTAC7 (200 nM)
HDPIC (200 nM)
SB-505124 (1 µM)
Time after washout (h)
0 0 0 0 4 4 4 4 4 4
p-SMAD3 pSer423/Ser425
Halo-SMAD3
SMAD3
Halo
GAPDH
K
PhosTAC7
FKBP12F36V (dTAG) binder
HaloTag binder

Figure S3: Characterization of HDPIC (HaloTag-dTAG proximity-inducing chimera). In relation to Fig. 2.

(**A**) Schematic of recruitment of a Halo-POI to a dTAG-phosphatase to mediate targeted Halo-POI dephosphorylation using bifunctional molecule HDPIC (HaloTag-dTAG proximity-inducing chimera).

(**B**) Structure of HDPIC.

(**C**) Cytotoxicity of HDPIC was measured using CellTox Green Assay (Promega) by treating wild-type (WT) U2OS or A549 cells with HDPIC at the indicated concentrations for 24 h. DMSO was included as a negative control and MG132 (20 µM, 24 h) or the lysis buffer provided in the assay kit used as a positive control. Fluorescence was then measured using a PHERAstar plate reader (ex: 480 nm em: 530 nm). Data represent n=3. Values are shown as a mean fluorescence reading normalized to DMSO controls ± SD.

(**D**) Engagement of Halo-POI by HDPIC was shown by band-shift of small (~35 kDa) artificial FLAG-NLS(nuclear localization signal)-Halo-HiBiT protein upon HDPIC treatment. U2OS cells stably expressing FLAG-NLS-Halo-HiBiT were treated for 2 h with the indicated concentrations of HDPIC or an equivalent volume of DMSO. Cells were lysed before extracts were resolved by SDS-PAGE, transferred to nitrocellulose membrane and immunoblotted with the indicated antibodies. Three independent experiments are shown together.

(**E**) Engagement of dTAG-POI by HDPIC was shown by means of a competition-style assay. HEK293 WT or HEK293 $^{dTAG/dTAG}$*PPP2CA* cells were treated for 24 h with DMSO, dTAG-13 PROTAC (100 nM), HDPIC (1 µM) or a combination of dTAG-13 and HDPIC. Cells were lysed and processed as in (**D**).

(**F**) U2OS cells stably expressing Halo-SMAD3 alone or in combination with dTAG-PPM1H were serum-starved (16 h) before 1 h stimulation with TGFβ (5 µg/L). 0 h samples were lysed at this point. For other samples, TGFβ stimulation was removed and cells were placed in fresh serum-free medium without TGFβ with DMSO, HDPIC (1 µM) or no treatment. Samples were then lysed and processed as in (**D**).

(**G**) Western blotting screening of multiple A549 ^{Halo}*SMAD3* knock-in clones, with the clone of interest, Clone 19, highlighted in red. Cells were lysed and processed as in (**D**).

(**H**) Confirmation of multiple knock-in clones, with clone 19 highlighted in red, by amplification of the target *SMAD3* genomic region through polymerase chain reaction. DNA from parental WT A549 cells was included as a negative control.

(**I**) Confirmation of insertion of knock-in at desired genomic locus by sequencing of the target locus in A549 $^{Halo/WT}$*SMAD3* clone 19 cells.

(**J**) To show effect of HDPIC on Halo-SMAD3 phosphorylation, A549 $^{Halo/WT}$*SMAD3* knock-in cells were serum-starved (16 h) prior to 1 h co-treatment with TGFβ (5 µg/L) and SB-505124 (1 µM) or DMSO. 0 h time points were lysed at this moment. For 4 h time points, TGFβ

stimulation was removed by washout and fresh serum-free medium without TGFβ was added to cells, along with DMSO, HDPIC (200 nM) or PhosTAC7 (200 nM) for 4 h. Samples were then lysed and processed as in (**D**).

(**K**) Structure of PhosTAC7.

Data are representative of n=3 independent experiments, except (**G-I**).

Figure S4

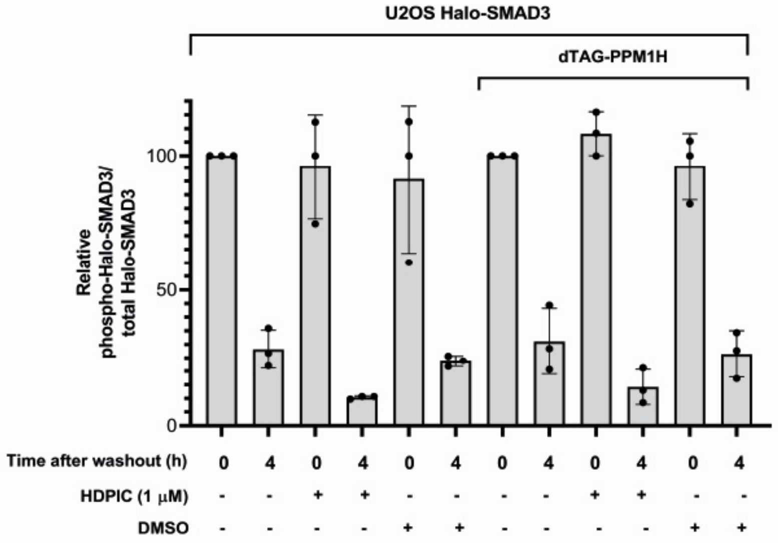

Figure S4: Quantification of relative Western blot signal intensities from biological repeats of Figure S3F. In relation to Fig. S3 and Fig. 2.

Quantification of n=3 independent experiments of Fig. S3F, showing mean ± SD phospho-dTAG-SMAD3/total dTAG-SMAD3 levels, relative to the 0 h sample for each cell line.

Figure S5

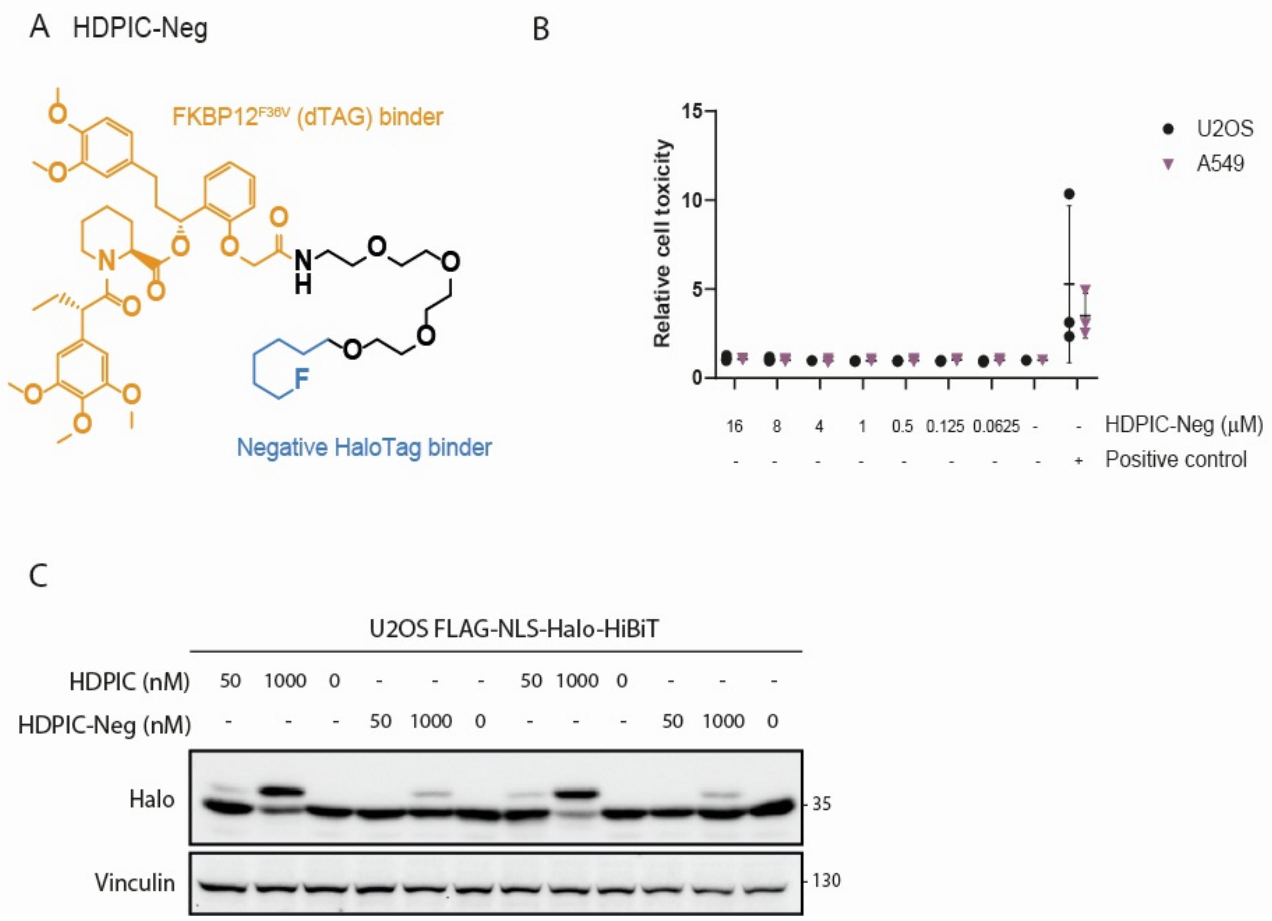

Figure S5: Characterization of HDPIC-Neg and identification of retained interaction with HaloTag at micromolar concentrations. In relation to Fig. S3 and Fig. 2.

(**A**) Structure of HDPIC-Neg.

(**B**) Cytotoxicity of HDPIC-Neg was measured using CellTox Green Assay (Promega) as in (**Fig. S3C**), in WT U2OS and A549 cells. Data represent n=3. Values are shown as a mean fluorescence reading normalized to DMSO controls ± SD.

(**C**) Engagement of Halo-POI by HDPIC-Neg was probed by a band-shift assay in U2OS FLAG-NLS-Halo-HiBiT cells following 2 h treatment with the indicated concentrations of HDPIC-Neg, HDPIC (active) or an equivalent volume of DMSO. Cells were lysed before extracts were resolved by SDS-PAGE, transferred to nitrocellulose membrane and immunoblotted with the indicated antibodies. Data are representative of n=3 independent experiments.

Figure S6

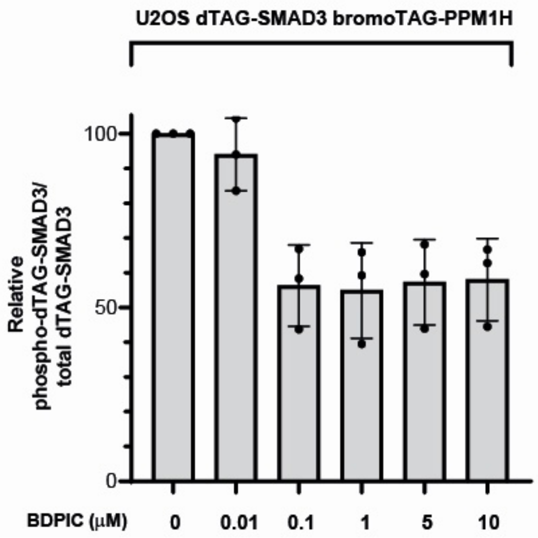

Figure S6: Quantification of relative Western blot signal intensities from biological repeats of Figure 3A. In relation to Fig. 3.

Quantification of n=3 independent experiments of Fig. 3A, showing mean ± SD phospho-dTAG-SMAD3/total dTAG-SMAD3 levels, relative to DMSO control treatment (lane 1).

Figure S7

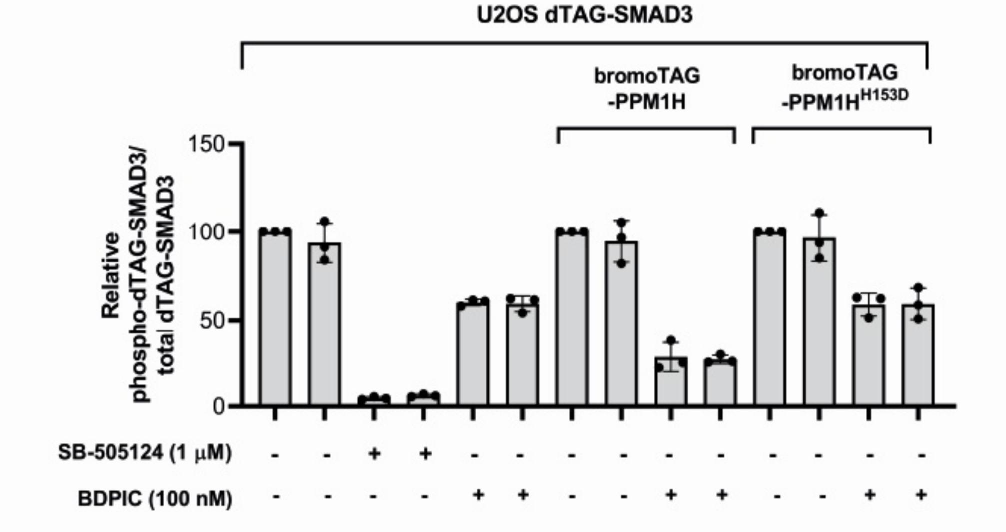

Figure S7: Quantification of relative Western blot signal intensities from biological repeats of Figure 4C. In relation to Fig. 4.

Quantification of n=3 independent experiments of Fig. 4C, showing mean ± SD phospho-dTAG-SMAD3/total dTAG-SMAD3 levels, relative to the first lane of DMSO treatment for each cell line.

Figure S8

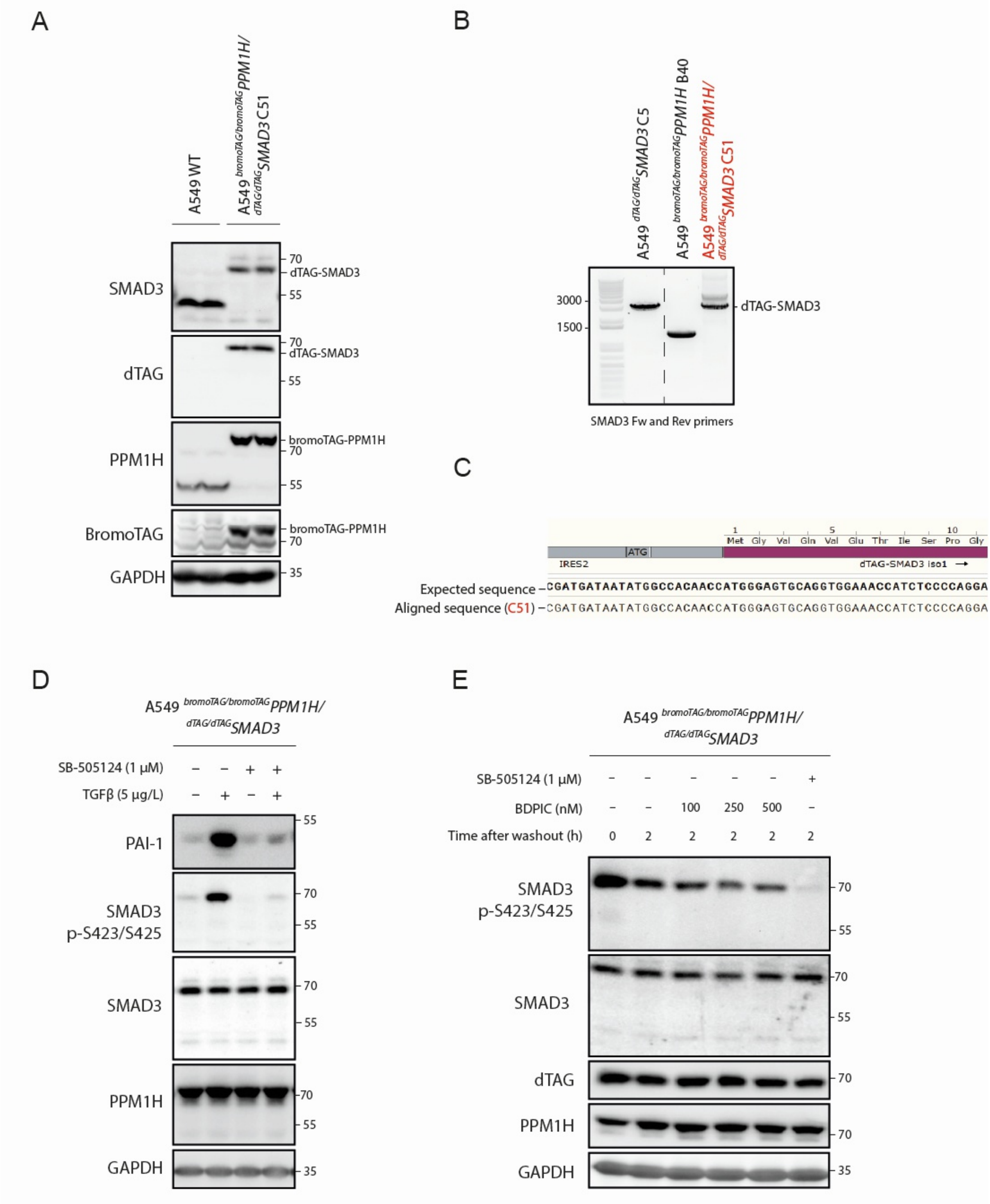
A
A549 WT
A549 bromoTAG/bromoTAG PPM1H/ dTAG/dTAG SMAD3 C51
SMAD3
dTAG
PPM1H
BromoTAG
GAPDH
70
dTAG-SMAD3
55
bromoTAG-PPM1H
35
B
A549 dTAG/dTAG SMAD3 C5
A549 bromoTAG/bromoTAG PPM1H B40
A549 bromoTAG/bromoTAG PPM1H/ dTAG/dTAG SMAD3 C51
3000
1500
dTAG-SMAD3
SMAD3 Fw and Rev primers
C
Met Gly Val Gln Val Glu Thr Ile Ser Pro Gly
ATG
IRES2
dTAG-SMAD3 iso1
Expected sequence – CGATGATAATATGGCCACAACCATGGGAGTGCAGGTGGAAACCATCTCCCCAGGA
Aligned sequence (C51) – CGATGATAATATGGCCACAACCATGGGAGTGCAGGTGGAAACCATCTCCCCAGGA
D
A549 bromoTAG/bromoTAG PPM1H/ dTAG/dTAG SMAD3
SB-505124 (1 µM) – – + +
TGFβ (5 µg/L) – + – +
PAI-1
SMAD3 p-S423/S425
SMAD3
PPM1H
GAPDH
E
A549 bromoTAG/bromoTAG PPM1H/ dTAG/dTAG SMAD3
SB-505124 (1 µM) – – – – – +
BDPIC (nM) – – 100 250 500 –
Time after washout (h) 0 2 2 2 2 2
SMAD3 p-S423/S425
SMAD3
dTAG
PPM1H
GAPDH

Figure S8: Generation and validation of A549 $^{bromoTAG/bromoTAG}$*PPM1H*/$^{dTAG/dTAG}$*SMAD3* knock-in cells. In relation to Fig. 5.

(**A**) Western blot of A549 wild-type (WT) and A549 $^{bromoTAG/bromoTAG}$*PPM1H*/$^{dTAG/dTAG}$*SMAD3* clone 51 cells. For Western blotting, cells were lysed before extracts were resolved by SDS-PAGE, transferred to nitrocellulose membrane and subjected to immunoblotting with the indicated antibodies.

(**B**) Confirmation of knock-in by amplification of the target *SMAD3* genomic region through polymerase chain reaction, with clone 51 highlighted in red. DNA from parental A549 $^{bromoTAG/bromoTAG}$*PPM1H* cells was included as a negative control while DNA from knock-in A549 $^{dTAG/dTAG}$*SMAD3* clone 5 cells was included as a positive control. Samples were all run on the same gel and non-relevant samples have been omitted from between samples of interest.

(**C**) Confirmation of insertion of knock-in at desired genomic locus by sequencing of the target locus in A549 $^{bromoTAG/bromoTAG}$*PPM1H*/$^{dTAG/dTAG}$*SMAD3* clone 51 cells.

(**D**) Validation that endogenous dTAG-SMAD3 in A549 $^{bromoTAG/bromoTAG}$*PPM1H*/$^{dTAG/dTAG}$*SMAD3* clone 51 cells retain endogenous function and is responsive to TGFβ stimulation and capable of subsequently increasing PAI-1 protein levels. Here, cells were stimulated with TGFβ (5 µg/L) or control and treated with SB-505124 or DMSO for 6 h prior to being processed as in (**A**).

(**E**) Preliminary dose response of BDPIC-mediated targeted dephosphorylation of dTAG-SMAD3 in A549 $^{bromoTAG/bromoTAG}$*PPM1H*/$^{dTAG/dTAG}$*SMAD3* cells. Following serum-starvation (16 h), cells were stimulated with TGFβ (5 µg/L) for 1 h. 0 h time points were lysed at this moment. For 2 h time points, TGFβ stimulation was removed by washout and fresh serum-free medium without TGFβ was added to cells, along with DMSO or the indicated concentrations of BDPIC for 2 h. SB-505124 was employed as a control. Cells were then lysed and processed as in (**A**).

Data in (**A**) and (**D**) are representative of n=3 independent experiments, other data are representative of n=1.

Figure S9

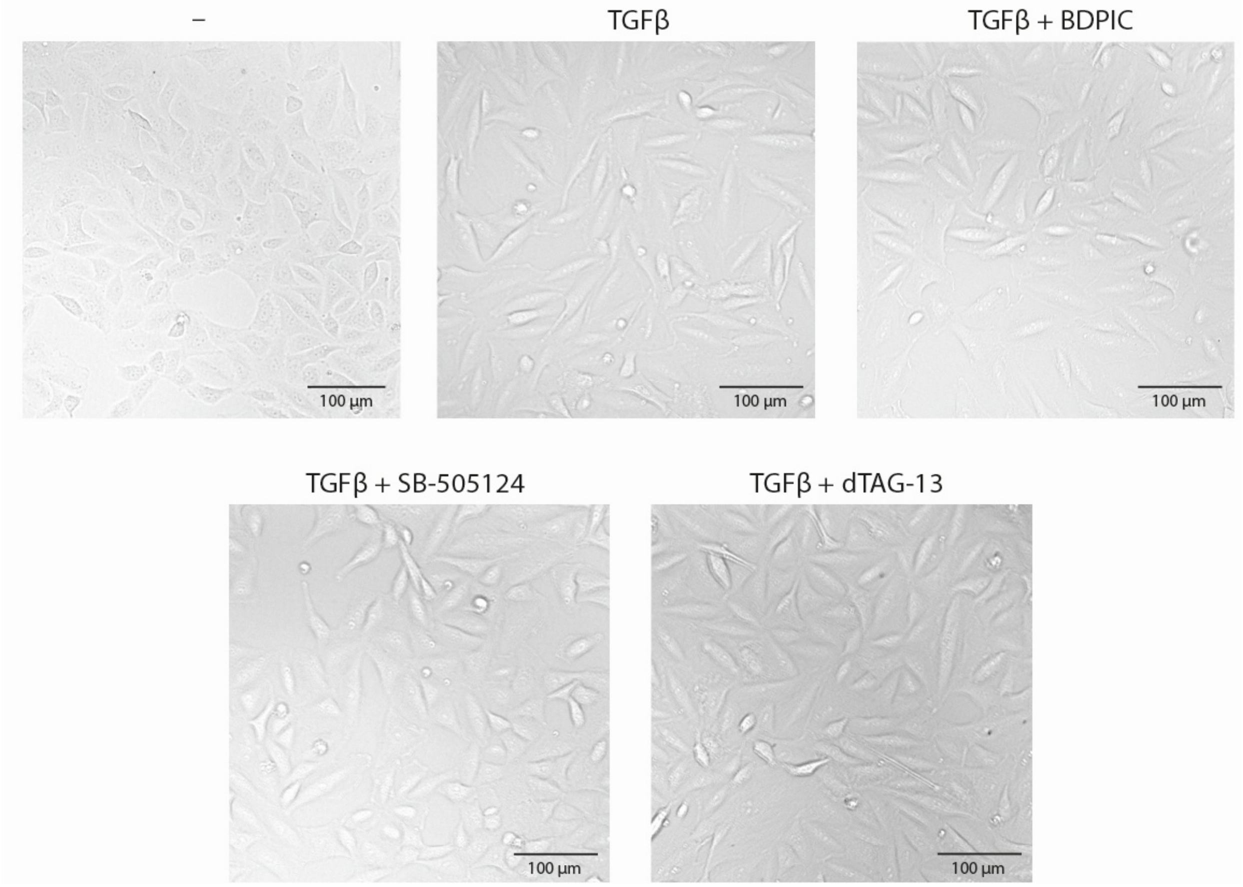

Figure S9: Bright-field images for EMT assay in A549 $^{\textit{bromoTAG/bromoTAG}}$*PPM1H*/$^{\textit{dTAG/dTAG}}$*SMAD3* knock-in cells. In relation to Fig. 6.

Representative bright-field microscopy images of serum-starved A549 $^{\textit{bromoTAG/bromoTAG}}$*PPM1H*/$^{\textit{dTAG/dTAG}}$*SMAD3* cells from Fig. 6B that were treated for 48 h with or without TGFβ (5 µg/L) and DMSO, BDPIC (250 nM), SB-505124 (1 µM) or dTAG-13 (100 nM) as indicated. The scale bars in the images represent 100 µm. Images are representative of n=3 independent experiments.

Methods S1: Chemistry methods: Related to STAR Methods and Figures 1, 2, 3, 4, 5 and 6.

Synthesis of BDPIC (bromoTAG-dTAG proximity-inducing chimera)

To ensure access of the full methodology for BDPIC synthesis, we include the identical detailed methodology presented here also in the manuscript by *Zhao et al* that was jointly submitted for publication in *iScience*.

Abbreviations:

AcOH	Acetic acid
DCE	1,2-Dichloroethane
DCM	Dichloromethane
DIC	*N*,*N* -Diisopropylcarbodiimide
DIPEA	*N*,*N*-Diisopropylethylamine
DMAP	4-(Dimethylamino)pyridine
DMF	*N,N*-Dimethylformamide
DMSO	Dimethyl sulfoxide
Et_3N	Triethylamine
EtOAc	Ethyl acetate
Et_3SiH	Triethylsilane
LiHMDS	Lithium bis(trimethylsilyl)amide
MeCN	Acetonitrile
MeOH	Methanol
NH_4Cl	Ammonium chloride
$Pd(OAc)_2$	Palladium(II) acetate
PyBOP	Benzotriazole-1-yl-oxy-tris-pyrrolidino-phosphonium hexafluorophosphate
TFA	Trifluoroacetic acid
THF	Tetrahydrofuran
T3P	Propanephosphonic acid anhydride

General comments

All chemicals were purchased from commercial vendors and used without further purification. Flash column chromatography and Prep HPLC were performed by using Buchi PrepChrom C-700, prepacked Buchi Sepacore Flash Cartridges and HPLC C18 column Gemini NY(RP)C18 110, 21.2×150 mm, 10 μn particle size. Details about the conditions for preparative HPLC are provided in the experimental procedures. NMR spectra were recorded on a Bruker Ascend 500 MHz. Chemical shifts are reported in parts per million referenced to

residual solvent peaks ($CDCl_3$= 7.26 ppm). Only the chemical shifts of the major rotamer are reported. The following abbreviations were used in reporting spectra, s (singlet), d (doublet), t (triplet), q (quartet), m (multiplet), dd (doublet of doublets), bs (broad signal). Low resolution mass spectra and analytical HPLC traces were recorded on an Agilent Technologies 1200 series HPLC connected to an Agilent Technologies 6130 quadrupole LC/MS, connected to an Agilent diode array detector. The column used was a Waters XBridge column (50 mm × 2.1 mm, 3.5 μm particle size), with a gradient from 5 % to 95% of acetonitrile in water (with 0.1 % of formic acid or aqueous ammonia solution) over 3 or 7 minutes. The flow rate was 0.7 mL/min.

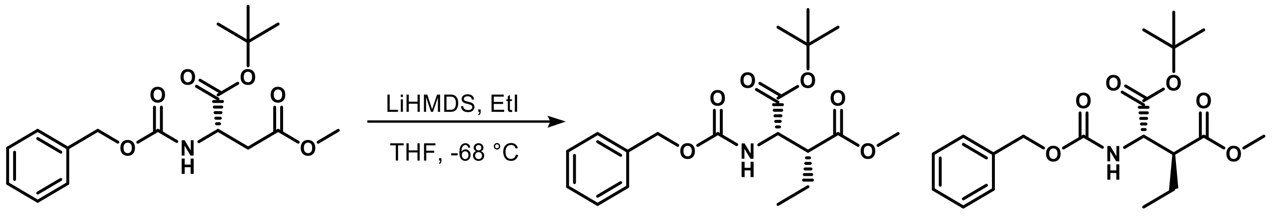

1 2 3

1-(tert-butyl) 4-methyl (2S,3R)-2-(((benzyloxy)carbonyl)amino)-3-ethylsuccinate (**2**) and 1-(tert-butyl) 4-methyl (2S,3S)-2-(((benzyloxy)carbonyl)amino)-3-ethylsuccinate (**3**)

To a solution of 1-(tert-butyl) 4-methyl ((benzyloxy)carbonyl)-L-aspartate **1** [1] (9.38 g, 27.80 mmol) in 250 mL of anhydrous THF was added dropwise 1.3 M solution of LiHMDS in THF (45 mL, 58.5 mmol) at -68°C.The reaction mixture was stirred at -68°C for 1h, then at -40°C for 1h, and cooled down again to -68°C. Iodoethane (5.6 mL, 10.86 g, 69.62 mmol) was then added dropwise and the reaction was stirred for 1h at -10°C, quenched with saturated NH_4Cl solution (75 mL) and extracted with EtOAc (2 x 100 mL). The organic layers were washed with brine (75 mL), combined, and concentrated. The crude residue (10 g) was purified by flash column chromatography on silica (200 g), gradient from petroleum spirit to 40% EtOAc in petroleum spirit to afford compound **2** (5.79 g, 15.84 mmol, 57%) and **3** (2.28g, 6.23 mmol, 22%).

2:^{1}H NMR (500 MHz, $CDCl_3$,): δ = 7.40-7.37 (m, 4H); 7.36-7.30 (m, 1H); 5.70 (d, J=9.3Hz, 1H); 5.15 (s, 2H); 4.55 (dd, J=4.0Hz, J=9.75Hz, 1H); 3.69 (s, 3H); 2.96 (ddd, J=4.0Hz, 1H); 1.85-1.73 (m, 1H); 1.64-1.55 (m, 1H); 1.45 (s, 9H); 1.01 (t, J=7.4Hz, 3H).

3: ^{1}H NMR (500 MHz, $CDCl_3$,): δ = 7.40-7.37 (m, 4H); 7.36-7.31 (m, 1H); 5.49 (d, J=7.2Hz, 1H); 5.12 (s, 2H); 4.56 (dd, J=5.0Hz, J=8.3Hz, 1H); 3.71 (s, 3H); 2.78-2.72 (m, 1H); 1.90-1.80 (m, 1H); 1.62-1.53 (m, 1H); 1.48 (s, 9H); 0.98 (t, J=7.2Hz, 3H).

$C_{19}H_{27}NNaO_6$, expected for $[M+Na]^+$ 388.17, found $[M+Na]^+$ 388.0

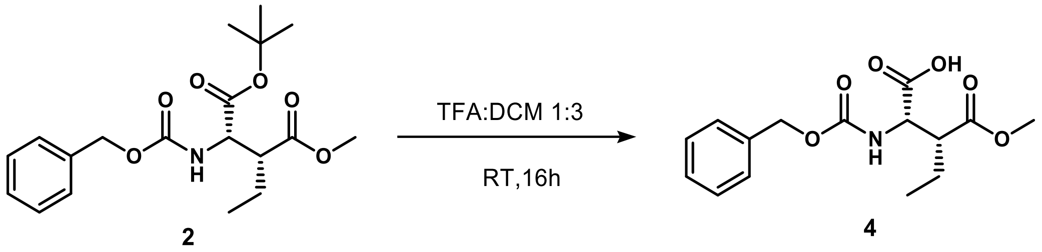

(2S,3R)-2-(((benzyloxy)carbonyl)amino)-3-(methoxycarbonyl)pentanoic acid (**4**)

To a solution of 1-(tert-butyl) 4-methyl (2S,3R)-2-(((benzyloxy)carbonyl)amino)-3-ethylsuccinate (**2**) (2.10 g, 5.75 mmol) in DCM (5 mL) was added solution of TFA in DCM (40 mL, 1:3 v/v) and the reaction mixture was stirred at RT for 16h and concentrated. Crude residue was purified by RP C18 flash column chromatography (SVP D40-RP18 25-40 μm 90 g), in a gradient of MeOH/H_2O (5% to 95%) to afford compound **4** (1.39 g, 4.50 mmol, 78%).
^{1}H NMR (500 MHz, $CDCl_3$,): δ = 7.40-7.33 (m, 5H); 5.89 (d, J=9.4Hz, 1H); 5.17 (s, 2H); 4.68 (dd, J=3.6Hz, J=9.4Hz, 1H); 3.73 (s, 3H); 3.08 (ddd, J=3.5Hz, 1H); 1.89-1.80 (m, 1H); 1.70-1.60 (m, 1H); 1.05 (t, J=7.4Hz, 3H).
$C_{15}H_{19}NNaO_6$, expected for $[M+Na]^+$ 332.11, found $[M+Na]^+$ 332.1

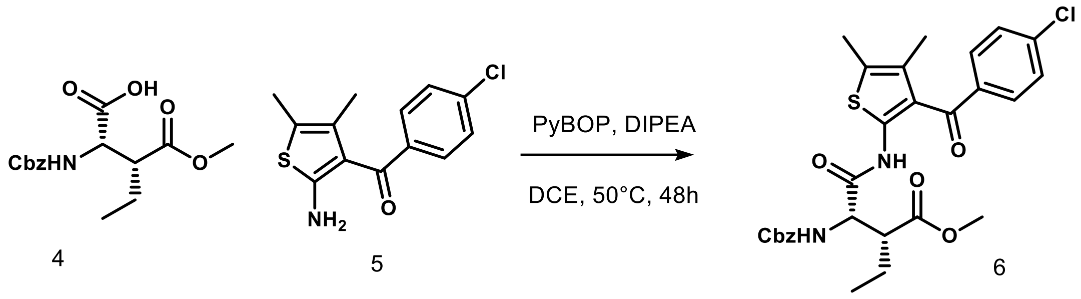

Methyl (2R,3S)-3-(((benzyloxy)carbonyl)amino)-4-((3-(4-chlorobenzoyl)-4,5-dimethylthiophen-2-yl)amino)-2-ethyl-4-oxobutanoate (**6**).

To a solution of **4** (1.53 g, 4.94 mmol) in DCE (45 mL) were added **5** [2] (1.31 g, 4.94 mmol), DIPEA (4.30 mL, 24.7 mmol) and PyBOP (6.42 g, 12.35 mmol) at RT. The reaction mixture was stirred at 50°C for 48 h, cooled to RT, diluted with water (25 mL) and extracted with DCM (2×50 mL). The organic layer was washed with brine (25 mL) and concentrated. The crude residue was purified by column chromatography on silica (160 g), gradient from petroleum spirit to 40% EtOAc in petroleum spirit to give crude material (1.57 g) which was further purified

by RP C18 flash column chromatography (SVP D40-RP18 25-40 µm 90 g) in a gradient of MeCN/H_2O (5% to 95%) to afford compound **6** (1.13 g, 2.02 mmol, 41%).

^{1}H NMR (500 MHz, $CDCl_3$,): δ = 11.74 (s,1H); 7.56(d, J=8.4Hz, 2H); 7.45 (d, J=8.4Hz,4H); 7.34-7.29 (m, 3H); 6.24(d, J=9.4Hz,1H); 5.30 (d, J=12.1Hz, 1H); 5.18 (d, J=12.1Hz,1H); 4.75 (dd, J=3.4Hz, J=9.6Hz, 1H); 3.68 (s, 3H); 3.34-3.27 (m, 1H); 2.27 (s, 3H); 1.89-1.79 (m, 1H); 1.71 (s, 3H); 1.67-1.59 (m, 1H); 1.05 (t, J=7.4Hz, 3H).

$C_{28}H_{29}ClN_2NaO_6S$, expected for $[M+Na]^+$ 579.13, found $[M+Na]^+$ 579.0

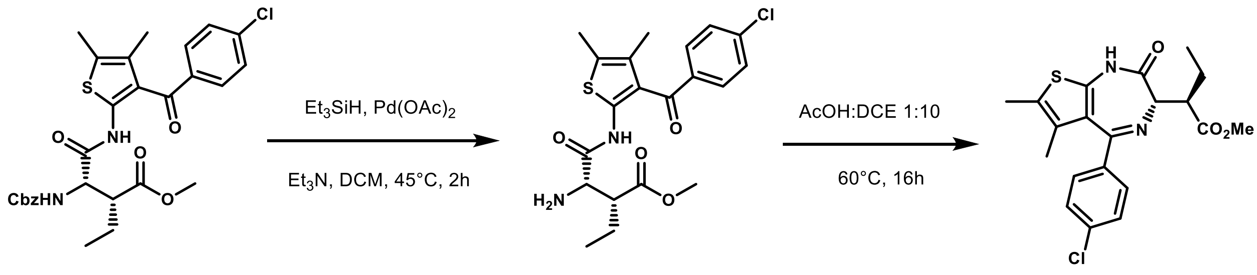

6 7 8

Methyl (R)-2-((S)-5-(4-chlorophenyl)-6,7-dimethyl-2-oxo-2,3-dihydro-1H-thieno[2,3-e][1,4]diazepin-3-yl)butanoate (**8**)

To a solution of **6** (0.98 g, 1.76 mmol) in DCM (18 mL) were added $Pd(OAc)_2$ (0.04 g, 0.17 mmol) and Et_3N (0.1 mL, 0.71 mmol) and the reaction was heated at 45°C for 5 min while triethylsilane (1.40 mL,1.01 g, 8.76 mmol) was added dropwise. The reaction mixture was further stirred at 45°C for 2 h, cooled to RT, diluted with DCM (20 mL), filtered through Celite, and concentrated. The crude residue of methyl(2R,3S)-3-amino-4-((3-(4-chlorobenzoyl)-4,5-dimethylthiophen-2-yl)amino)-2-ethyl-4-oxobutanoate (**7**) was used in the next step without further purification.

$C_{20}H_{24}ClN_2O_4S$, expected for $[M+H]^+$ 423.11, found $[M+H]^+$ 423.1.

To a solution of **7** (crude residue 1.1 g) in DCE (10 mL) 10% of acetic acid was added and the reaction mixture was kept at 60°C for 16h and concentrated. The crude residue was purified by flash column chromatography on silica (160 g), gradient from petroleum spirit to 50% EtOAc in petroleum spirit to afford compound **8** (0.37g, 0.91 mmol, 51% for two steps) as yellow shiny crystals. Enantiomeric purity of **8** was determined as 99% *ee* on chiral column CHIRALPAC IA (20x250 mm,5 µn particle size, DAICEL), 20% EtOAc in heptane, 220 nm, v=6 mL/min with t_R=15.1 min.

^{1}H NMR (500 MHz, $CDCl_3$,): δ = 8.70 (br s,1H); 7.34 (m, 4H); 3.88 (d, J=10.4Hz, 1H); 3.83 (s, 3H); 3.66 (ddd, J=4.5Hz, 1H); 2.31 (s, 3H); 1.97-1.89 (m, 1H); 1.62 (s, 3H); 1.63-1.57 (m, 1H); 1.03 (t, J=7.5Hz, 3H).

$C_{20}H_{22}ClN_2O_3S$, expected for $[M+H]^+$ 405.10, found $[M+H]^+$ 405.1
$[\alpha]^{20}_D$ = +44.1 (c 0.5, $CHCl_3$)

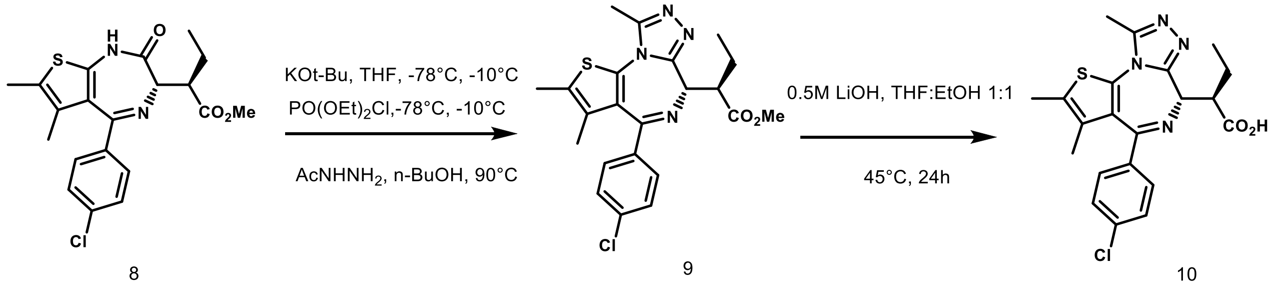

Methyl (R)-2-((S)-4-(4-chlorophenyl)-2,3,9-trimethyl-6H-thieno[3,2-f][1,2,4]triazolo[4,3-a][1,4]diazepin-6-yl)butanoate (**9**).

The title compound was obtained as described in [3] starting from **8** (0.25 g, 0.61 mmol) in 73% yield (0.20 g, 0.45mmol) as colorless crystals. Enantiomeric purity of **9** was determined as 99% *ee* on chiral column CHIRALPAC IA (20x250 mm,5 μn particle size, DAICEL), 20% EtOAc in heptane, 220 nm, v=6 mL/min with t_R=19.2 min.

^{1}H NMR (500 MHz, $CDCl_3$,): δ = 7.34 (m, 4H); 4.26 (d,J=10.9Hz,1H); 4.01 (ddd, J=3.8Hz, 10.7Hz, 10.9Hz, 1H); 3.87 (s, 3H); 2.69 (s, 3H); 2.44 (s, 3H); 2.25-2.15 (m, 1H); 1.70 (s, 3H); 1.74-1.63 (m, 1H); 1.04 (t, J=7.4Hz, 3H).
^{13}C NMR (126 MHz, $CDCl_3$) δ = 175.4, 163.2, 154.45,149.9, 149.8, 136.8, 136.6, 132.1, 130.9, 130.5, 129.8, 128.7, 59.4, 51.6, 49.7, 23.3, 14.5, 13.2, 11.9, 11.7
$C_{22}H_{24}ClN_4O_2S$, expected for $[M+H]^+$ 443.13, found $[M+H]^+$ 443.1
$[\alpha]^{20}_D$ = +70.1 (c 0.5, $CHCl_3$)

To a solution of **9** (0.20 g, 0.45 mmol) in a mixture of THF:EtOH 1:1 (5 mL) was added 0.5M solution of LiOH (2.7 mL, 1.35 mmol) and the mixture was stirred at 45°C for 24 h, cooled to RT, acidified with 1M HCl (1.5 mL, 1.5 mmol), and concentrated. To a crude residue was added water (5 mL) and the precipitate was collected on sintered glass filter. It was purified by RP C18 flash column chromatography (SVP D40-RP18 25-40 μm 90 g), in a gradient of $MeCN/H_2O$ (5% to 95%) to afford compound **10** (0.115 g, 0.26 mmol, 59%).
^{1}H NMR (500 MHz, $CDCl_3$,): δ = 7.44 (d, J=8.6Hz, 2H); 7.36(d, J-8.6Hz, 2H); 4.25 (d, J=6.1, 1H); 3.73-3.63 (m, 1H); 2.71 (s, 3H); 2.46 (s, 3H); 2.12-2.02 (m, 1H); 2.00-1.90 (m, 1H); 1.74 (s, 3H); 1.12 (t, J=7.4Hz, 3H).

^{13}C NMR (126 MHz, $CDCl_3$) δ = 174.8, 164.7, 154.8,150.0, 137.4, 135.8, 132.4, 131.4, 131.2, 130.1, 128.8, 58.0, 48.4, 23.8, 14.7, 13.2, 11.8

$C_{21}H_{22}ClN_4O_2S$, expected for $[M+H]^+$ 429.12, found $[M+H]^+$ 429.1
$[\alpha]^{20}_D$ = +103.5 (c 0.5, $CHCl_3$)

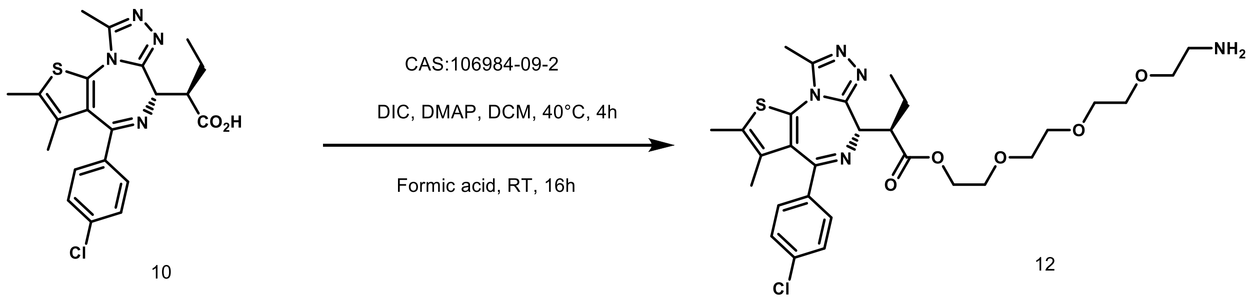

2,2-dimethyl-4-oxo-3,8,11,14-tetraoxa-5-azahexadecan-16-yl(R)-2-((S)-4-(4-chlorophenyl)-2,3,9-trimethyl-6H-thieno[3,2-f][1,2,4]triazolo[4,3-a][1,4]diazepin-6-yl)butanoate (**11**).

To a solution of **10** (0.030 g, 0 07 mmol) in DCM (2 mL) was added DIC (0.043 mL, 0.035g, 0.277 mmol), DMAP (0.011 g, 0.09 mmol) and the mixture was stirred at RT for 30 min. A solution of t-butyl-N-(2-(2-(2-(2-hydroxyethoxy)ethoxy)ethoxy)ethyl)carbamate (0.082g, 0.28 mmol) in DCM (0.5 mL) was then added and the reaction was heated at 40°C for 4 h and concentrated. The crude residue (0.15 g) was purified on HPLC RP C18 column with gradient $MeCN/H_2O$ (5% to 95%) to give **11** (0.028 g, 0.039 mmol, 56%) as a solid.
^1H NMR (500 MHz, $CDCl_3$,): δ = 7.36 (d, J=8.6Hz, 2H); 7.33(d, J-8.6Hz, 2H); 5.03 (br s, 1H); 4.49-4.37 (m, 2H); 4.27 (d, J=10.8Hz, 1H); 4.02 (ddd, J=3.6Hz, 1H); 3.86-3.78 (m, 2H); 3.74-3.70 (m, 2H); 3.68-3.61 (m, 6H); 3.55 (t, J=5.2Hz, 2H); 3.35-3.30 (m, 2H); 2.68 (s, 3H); 2.44 (s, 3H); 2.25-2.16 (m, 1H), 1.76-1.66 (m, 1H); 1.71 (s, 3H); 1.46 (s 9H); 1.06 (t, J=7.4Hz, 3H).
$C_{34}H_{47}ClN_5O_7S$, expected for $[M+H]^+$ 704.29, found $[M+H]^+$ 704.3

2-(2-(2-(2-aminoethoxy)ethoxy)ethoxy)ethyl(R)-2-((S)-4-(4-chlorophenyl)-2,3,9-trimethyl-6H-thieno[3,2-f][1,2,4]triazolo[4,3-a][1,4]diazepin-6-yl)butanoate (**12**)
A solution of **11** (0.028 g, 0.039 mmol) in formic acid (1 mL) was kept at RT for 16 h. The volatiles were removed in vacuo to yield **12** (0.028 g, 0.039 mmol), the crude residue was used in the next step without further purification.

$C_{29}H_{39}ClN_5O_5S$, expected for $[M+H]^+$ 604.24, found $[M+H]^+$ 604.2

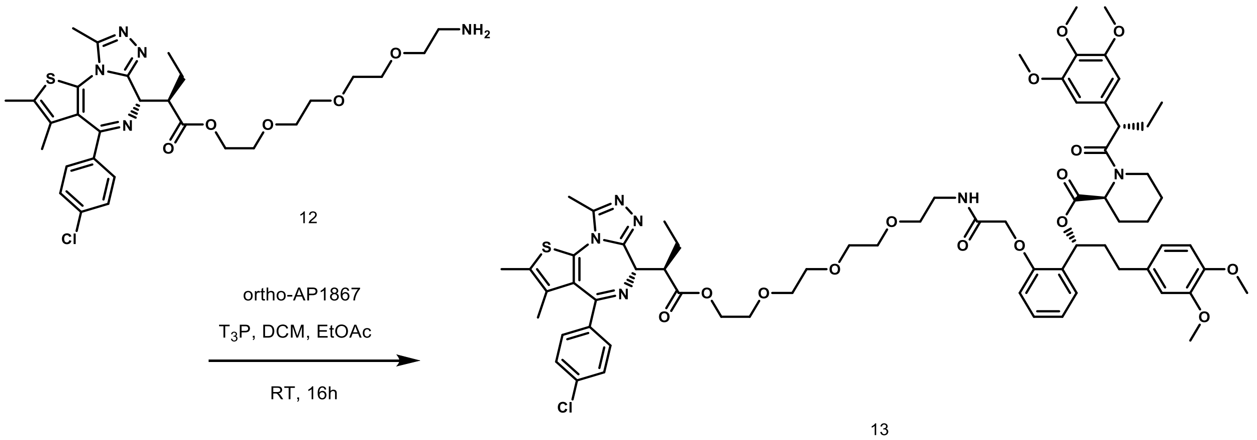

(R)-1-(2-(((R)-17-((S)-4-(4-chlorophenyl)-2,3,9-trimethyl-6H-thieno[3,2-f][1,2,4]triazolo[4,3-a][1,4]diazepin-6-yl)-2,16-dioxo-6,9,12,15-tetraoxa-3-azanonadecyl)oxy)phenyl)-3-(3,4-dimethoxyphenyl)propyl (S)-1-((S)-2-(3,4,5-trimethoxyphenyl)butanoyl)piperidine-2-carboxylate (**13**) (**BDPIC**).

To a solution of acid **ortho-AP1867** [4] (0.027 g, 0.038 mmol) in DCM (0.5 mL) were added a solution of **12** (0.028g, 0.038 mmol) in DCM (1 mL), DIPEA (0.093 mL, 0.53 mmol) and 50% solution of T3P in ethyl acetate at RT. The mixture was stirred at RT for 16 h, diluted with water (2 mL) and extracted with EtOAc (20 mL). The organic layer was washed with brine (5 mL) and concentrated. The crude residue was purified on HPLC RP C18 column with gradient MeCN/H_2O (5% to 95%) to give **13 (BDPIC)** (0.021 g, 0.016 mmol, 42%) as colorless powder. ^{1}H NMR (500 MHz, $CDCl_3$, mixture of rotamers, only peaks of the major rotamer are reported): δ = 7.42-7.37 (m, 1H); 7.36 (d, J=8.6Hz, 2H); 7.32 (d, J-8.6Hz, 2H); 7.22 (dt, J=1.7Hz, J=7.8Hz, 1H); 7.10-6.99 (m, 1H); 6.89 (t, J=7.6Hz,1H); 6.86-6.76 (m, 2H); 6.73-6.69 (m, 1H); 6.68 (s, 1H); 6.52-6.41 (m, 2H); 6.49 (s, 1H); 6.17 (dd, J=1.8Hz, J=6.0Hz, 1H); 5.51 (d, J=4.6Hz, 1H); 4.67-4.56 (m, 1H); 4.50 (ABq, J=14.8Hz, 2H); 4.47-4.34 (m, 2H); 4.27 (d, J=10.8Hz, 1H); 4.00 (ddd, J=3.6Hz, 1H); 3.88-3.84 (m, 12H); 3.80 (s, 3H); 3.70 (s, 3H); 3.69-3.64 (m, 2H); 3.62-3.51 (m, 11H); 3.50-3.40 (m, 2H); 2.68 (s, 3H); 2.69-2.46 (m, 2H); 2.43 (s, 3H); 2.33-1.93 (m, 6H); 1.82-1.73 (m, 1H); 1.72-1.64 (m, 1H); 1.70 (s, 3H); 1.05 (t, J=7.4Hz, 3H); 0.90 (t, J=7.2Hz, 3H).

$C_{67}H_{84}ClN_6O_{15}S$, expected for $[M+H]^+$ 1279.54, found $[M+H]^+$ 1279.6

Synthesis of HDPIC (HaloTag-dTAG proximity-inducing chimera) and HDPIC-Neg

Abbreviations:

DCM Dichloromethane

DIPEA *N*,*N*-Diisopropylethylamine

DMF *N,N*-Dimethylformamide

EtOAc Ethyl acetate

HATU 1-[Bis(dimethylamino)methylene]-1*H*-1,2,3-triazolo[4,5-*b*]pyridinium 3-oxid hexafluorophosphate

MeCN Acetonitrile

MeOH Methanol

THF Tetrahydrofuran

TFA Trifluoroacetic acid

General comments

All chemicals were purchased from commercial vendors and used without further purification. Ortho-AP1867 were prepared as described in [4].

Flash column chromatography and Prep HPLC were performed by using Buchi PrepChrom C-700, prepacked Buchi Sepacore Flash Cartridges and HPLC C18 column Gemini NY(RP)C18 110, 21.2×150 mm, 10 µn particle size. Details about the conditions for preparative HPLC are provided in the experimental procedures.

NMR spectra were recorded on a Bruker Ascend 500 MHz. Chemical shifts are reported in parts per million referenced to residual solvent peaks (CDCl3= 7.26 ppm). Only the chemical shifts of the major rotamer are reported. The following abbreviations were used in reporting spectra, s (singlet), d (doublet), t (triplet), q (quartet), m (multiplet), dd (doublet of doublets), bs (broad signal). Low resolution mass spectra and analytical HPLC traces were recorded on an Agilent Technologies 1200 series HPLC connected to an Agilent Technologies 6130 quadrupole LC/MS, connected to an Agilent diode array detector. The column used was a Waters XBridge column (50 mm × 2.1 mm, 3.5 µm particle size), with a gradient from 5 % to 95% of acetonitrile in water (with 0.1 % of formic acid) over 3 or 7 minutes. The flow rate was 0.7 mL/min.

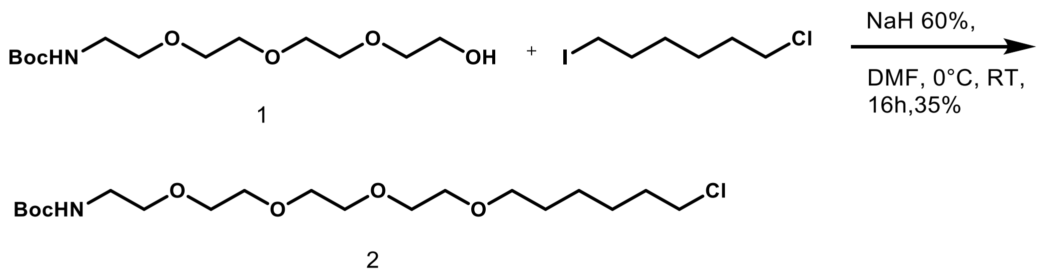

Synthesis of tert-butyl (18-chloro-3,6,9,12-tetraoxaoctadecyl)carbamate **2**

To a solution of tert-butyl (2-(2-(2-(2-hydroxyethoxy)ethoxy)ethoxy)ethyl)carbamate **1** (1.46 g, 5.0 mmol) in anhydrous DMF (20 mL) was added sodium hydride 60% in mineral oil (0.24g, 6.0 mmol) at 0 °C and the mixture was stirred at 0 °C for 1 hour followed by addition of a solution of 1-chloro-6-iodohexane (1.35 g, 5.5 mmol) in anhydrous DMF (5 ml). Reaction mixture was stirred at RT overnight, diluted with water (25 ml) and extracted with EtOAc (50 ml). Organic layer washed with brine (20 ml) and concentrated. The crude residue was purified by column chromatography on silica (80 g), gradient from petroleum spirit to 100% EtOAc to yield **2** (0.75 g, 1.82 mmol, 35%).

^{1}H NMR (400 MHz, $CDCl_3$) δ = 5.04 (bs, 1H), 3.70-3.63 (m, 10 H); 3.62-3.58 (m, 2H), 3.58-3.52 (m, 4H); 3.48 (t, 2H), 3.33 (q, 2H); 1.84-1.75 (m, 2H), 1.65-1.57(m, 4H), 1.47 (s, 9H); 1.44-1.36 (m, 2H).

$C_{19}H_{38}ClNO_6$, expected for $[M+H]^+$ 412.25, found $[M\text{-}Boc+H]^+$ 312.

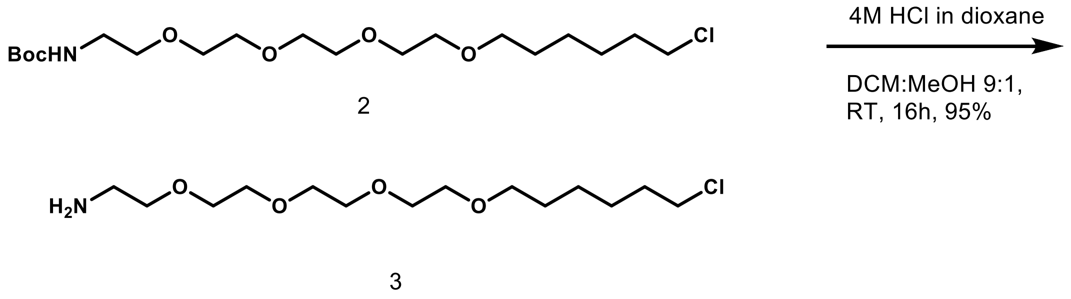

Synthesis of 18-chloro-3,6,9,12-tetraoxaoctadecan-1-amine hydrochloride **3**

To a solution of the **2** (0.75g, 1.82 mmol) in a mixture of DCM:MeOH 9:1 (10 mL) was added 4 M solution of HCl in dioxane (1.82 ml, 7.28 mmol) and the mixture was stirred at RT for 16 h. The reaction mixture was concentrated, crude residue of **3** (0.63 g, 1.80 mmol, 99%) as HCl salt used into next step without further purification.

^{1}H NMR (400 MHz, MeOD-d4) δ = 3.74 (t, J=5.06 Hz, 2H), 3.72-3.63 (m, 10 H); 3.63-3.59 (m, 2H), 3.57 (t, J=6.5 Hz, 2H); 3.51 (t, J=6.5 Hz,2H), 3.14 (t, J=5.06 Hz, 2H); 1.83-1.75 (m, 2H), 1.65-1.57(m, 2H), 1.54-1.38 (m, 4H).

$C_{14}H_{30}ClNO_4$, expected for $[M+H]^+$ 312.19, found $[M+H]^+$ 312.

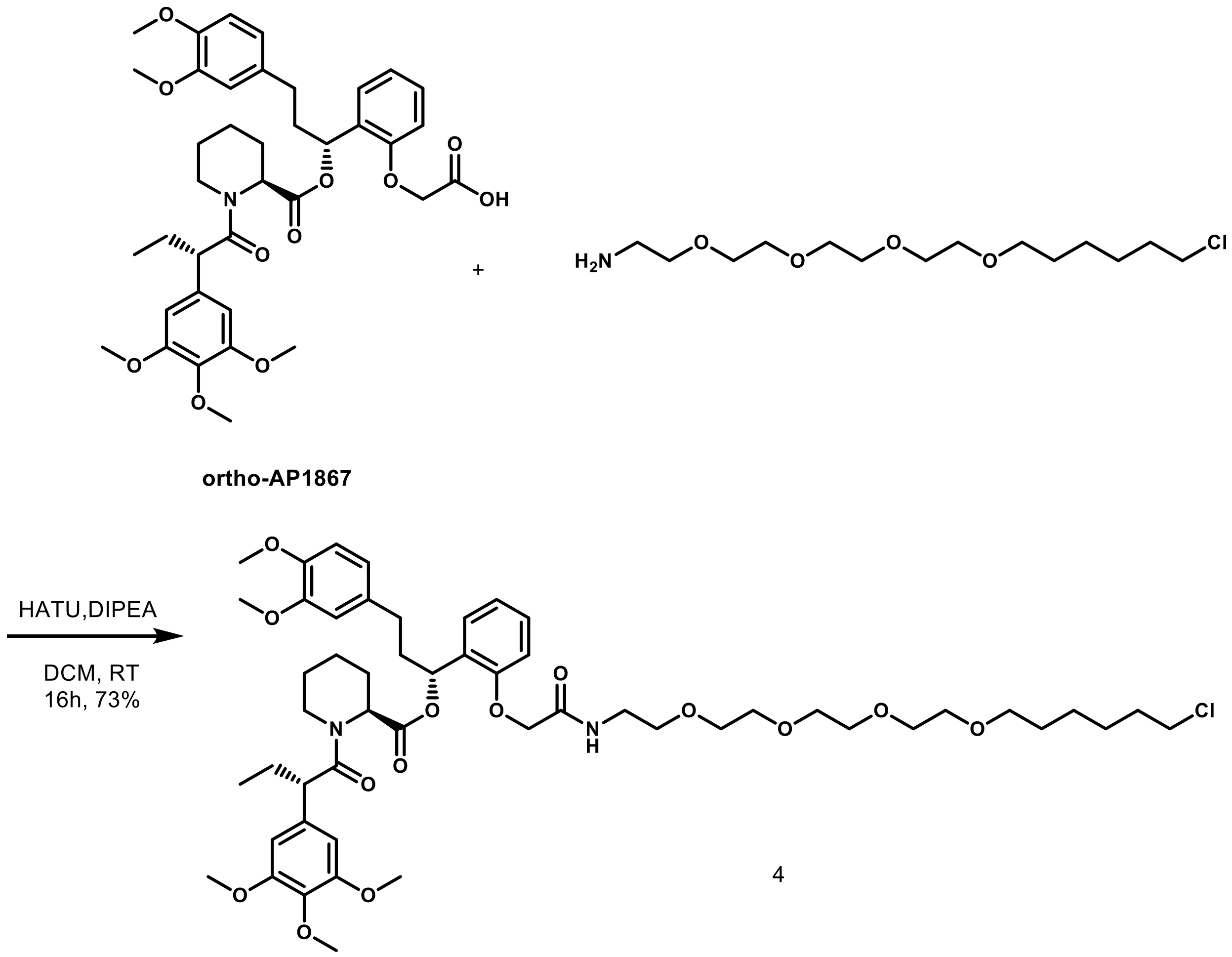

Synthesis of (R)-1-(2-((21-chloro-2-oxo-6,9,12,15-tetraoxa-3-azahenicosyl)oxy)phenyl)-3-(3,4-dimethoxyphenyl)propyl (S)-1-((S)-2-(3,4,5-trimethoxyphenyl)butanoyl)piperidine-2-carboxylate **4** (**HDPIC**)

To a solution of acid **ortho-AP1867** (0.08 g, 0.115 mmol) in DCM (5 mL) were added **3** (0.044g, 0.126 mmol), DIPEA (0.1 mL, 0.575 mmol) followed by addition of HATU (0.065 g, 0.172 mmol) at RT. The reaction mixture was stirred at RT for 16 h and concentrated. The

crude residue was purified by column chromatography on silica (16 g), gradient from DCM to 100% EtOAc, then to 20% of MeOH in EtOAc. The obtained crude material (0.09 g) was further purified on HPLC RP C18 column with gradient MeCN/H_2O (5% to 95%) to give **4** (**HDPIC**) (0.084 g, 0.085 mmol, 73%) as a solid.

^{1}H NMR (500 MHz, $CDCl_3$, mixture of rotamers, only peaks of the major rotamer are reported): δ = 7.40 (t, J=5.5Hz, 1H); 7.22 (dt, J=1.5Hz, J=7.8Hz, 1H); 7.19-7.15 (m, 1H); 7.09-7.06 (m, 1H); 6.91-6.87 (m, 1H); 6.84-6.77 (m, 2H); 6.73-6.66 (m, 1H); 6.69 (s, 1H); 6.49 (s, 1H); 6.51-6.42 (m, 1H); 6.41-6.32 (m, 1H); 6.17 (t, J=6.8Hz, 1H); 5.51(d, J=4.4 Hz, 1H); 4.66-4.44 (m, 3H); 3.90-3.85 (m, 12H); 3.80 (s, 2H); 3.71 (s, 3H); 3.64-3.52 (m, 20H); 3.46 (t, J=6.5 Hz, 2H); 2.71-2.44 (m, 3H); 2.32-1.93 (m, 4H); 1.83-1.75 (m, 2H); 1.64-1.55 (m, 2H); 1.50-1.34 (m, 4H); 0.90 (t, J=7.2Hz, 3H).

$C_{52}H_{75}ClN_2O_{14}$, expected for $[M+H]^+$ 987.50, found $[M+H]^+$ 988, 989

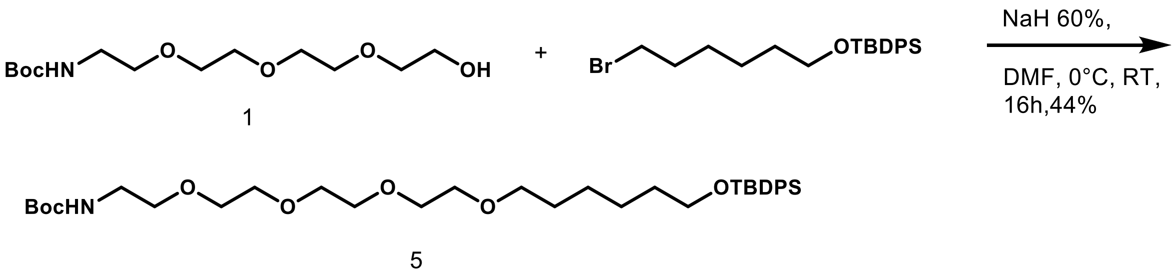

Synthesis of tert-butyl (2,2-dimethyl-3,3-diphenyl-4,11,14,17,20-pentaoxa-3-siladocosan-22-yl)carbamate (**5**)

To a solution of tert-butyl (2-(2-(2-(2-hydroxyethoxy)ethoxy)ethoxy)ethyl)carbamate **1** (0.89 g, 3.05 mmol) in anhydrous DMF (15 mL) was added sodium hydride 60% in mineral oil (0.146g, 3.66 mmol) at 0 °C and the mixture was stirred at 0 °C for 1 hour followed by addition of a solution of ((6-bromohexyl)oxy)(tert-butyl)diphenylsilane (1.27 g, 3.05 mmol) in anhydrous DMF (3 ml). Reaction mixture was stirred at RT overnight, diluted with water (20 ml) and extracted with EtOAc (50 ml). Organic layer washed with brine (20 ml) and concentrated. The crude residue was purified by column chromatography on silica (80 g), gradient from petroleum spirit to 50% EtOAc in petroleum spirit to yield **5** (0.85 g, 1.34 mmol, 44%).

^{1}H NMR (500 MHz, $CDCl_3$) δ = 7.68 (d, J=7.7Hz,4H); 7.46-7.37 (m, 6H); 5.09 (bs, 1H); 3.69-3.58 (m, 14 H); 3.56 (t, J=5.0Hz, 2H); 3.45 (t, J=6.8Hz, 2H); 3.33 (q, 2H); 1.64-1.54 (m, 4H); 1.46 (s, 9H); 1.42-1.27 (m, 4H); 1.06 (s, 9H).

$C_{35}H_{57}NO_7Si$, expected for $[M+Na]^+$ 654.38, found $[M+Na]^+$ 654.4.

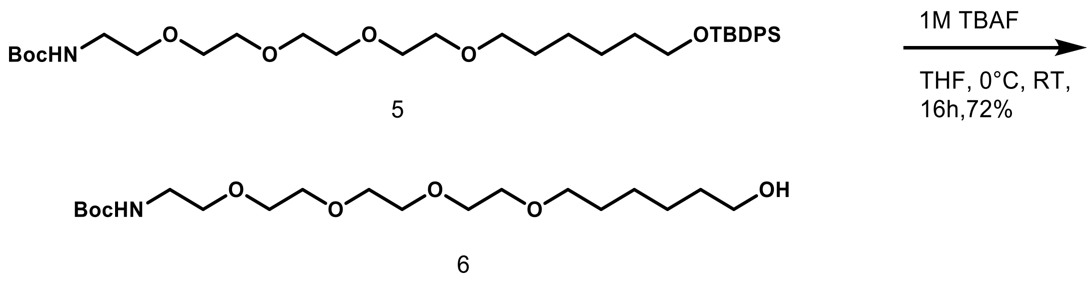

Synthesis of tert-butyl (18-hydroxy-3,6,9,12-tetraoxaoctadecyl)carbamate (**6**)

To a solution of **5** (0.85 g, 1.34 mmol) in THF (5 ml) was added 1M solution of TBAF in THF (1.47 ml, 1.47 mmol) at 0 °C and the mixture was stirred at RT for overnight. Water (5 ml) was added and extracted with EtOAc twice, organic layer washed with brine and concentrated. The crude residue was purified by column chromatography on silica (40g), gradient from DCM to 10% MeOH in DCM gave **6** (0.38 g, 0.965 mmol, 72%).

^{1}H NMR (500 MHz, $CDCl_3$) δ = 5.15 (bs, 1H); 3.70-3.59 (m, 14 H); 3.56 (t, J=5.0 Hz, 2H); 3.48 (t, J=6.6 Hz, 2H); 3.33 (q, 2H); 1.64-1.56 (m, 4H); 1.46 (s, 9H); 1.42-1.38 (m, 4H).

$C_{19}H_{39}NO_7$, expected for $[M+H]^+$ 394.28, found $[M\text{-}Boc+H]^+$ 294.3.

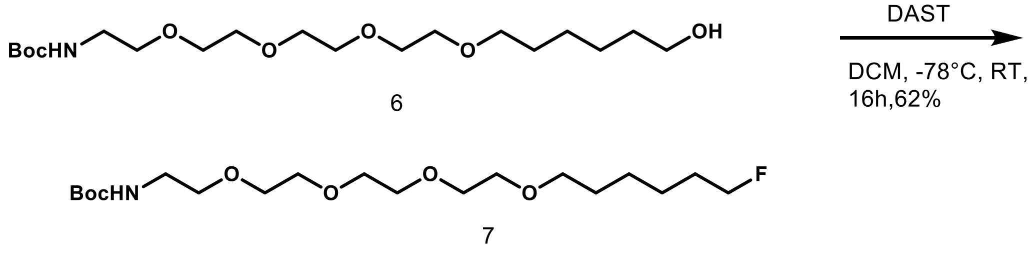

Synthesis of tert-butyl (18-fluoro-3,6,9,12-tetraoxaoctadecyl)carbamate (**7**)

To a solution of diethylaminosulfur trifluoride (DAST) (0.089 g, 0.072 ml, 0.552 mmol) in DCM (0.5 mL) was added a solution of **6** (0.087 g, 0.221 mmol) in DCM (1 mL) at -70 °C.
The mixture was stirred for 12 h at room temperature, quenched with saturated NaHCO3 (2 mL) and extracted with DCM (10 ml). The organic layer washed with brine and concentrated. The crude residue was purified by column chromatography on silica (12 g), gradient from DCM to 10% MeOH in DCM gave **7** (0.055g, 0.139 mmol, 62%).

^{1}H NMR (500 MHz, $CDCl_3$) δ = 5.09 (bs, 1H); 4.45 (dt, J=6.2 Hz, J=47.4 Hz, 2H); 3.71-3.58 (m, 10 H); 3.56 (t, J=5.0 Hz, 2H); 3.48 (t, J=6.6 Hz, 2H); 3.33 (m, 2H); 3.23 (m, 2H); 1.65-1.58 (m, 4H); 1.46 (s, 9H); 1.43-1.39 (m, 4H).

^{19}F NMR: δ = - 218.1

$C_{19}H_{38}FNO_6$, expected for $[M+H]^+$ 396.28, found $[M\text{-}Boc+H]^+$ 296.3.

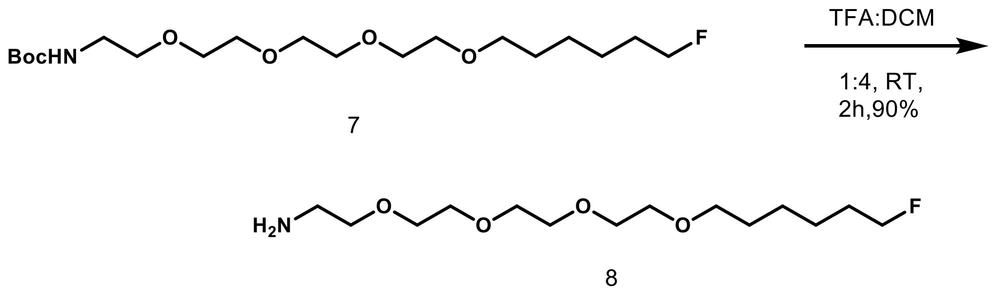

Synthesis of 18-fluoro-3,6,9,12-tetraoxaoctadecan-1-amine (**8**)

To a solution of **7** (0.053g, 0.134 mmol) in DCM (2ml) was added TFA (0.5 ml) at room temperature and reaction mixture was stirred at RT for 2 hours, concentrated and crude residue of **8** (0.050 g, 0.122mmol) as a TFA salt used into next step without further purification.

^{1}H NMR (500 MHz, $CDCl_3$) δ = 7.69 (bs, 2H); 4.46 (dt, J=6.2 Hz, J=47.4 Hz, 2H); 3.90-3.50 (m, 16 H); 3.21-3.14 (m, 2H); 1.65-1.38 (m, 8H).

^{19}F NMR: δ = - 75.1; -76.0

$C_{14}H_{30}FNO_4$, expected for $[M+H]^+$ 296.22, found $[M+H]^+$ 296.2.

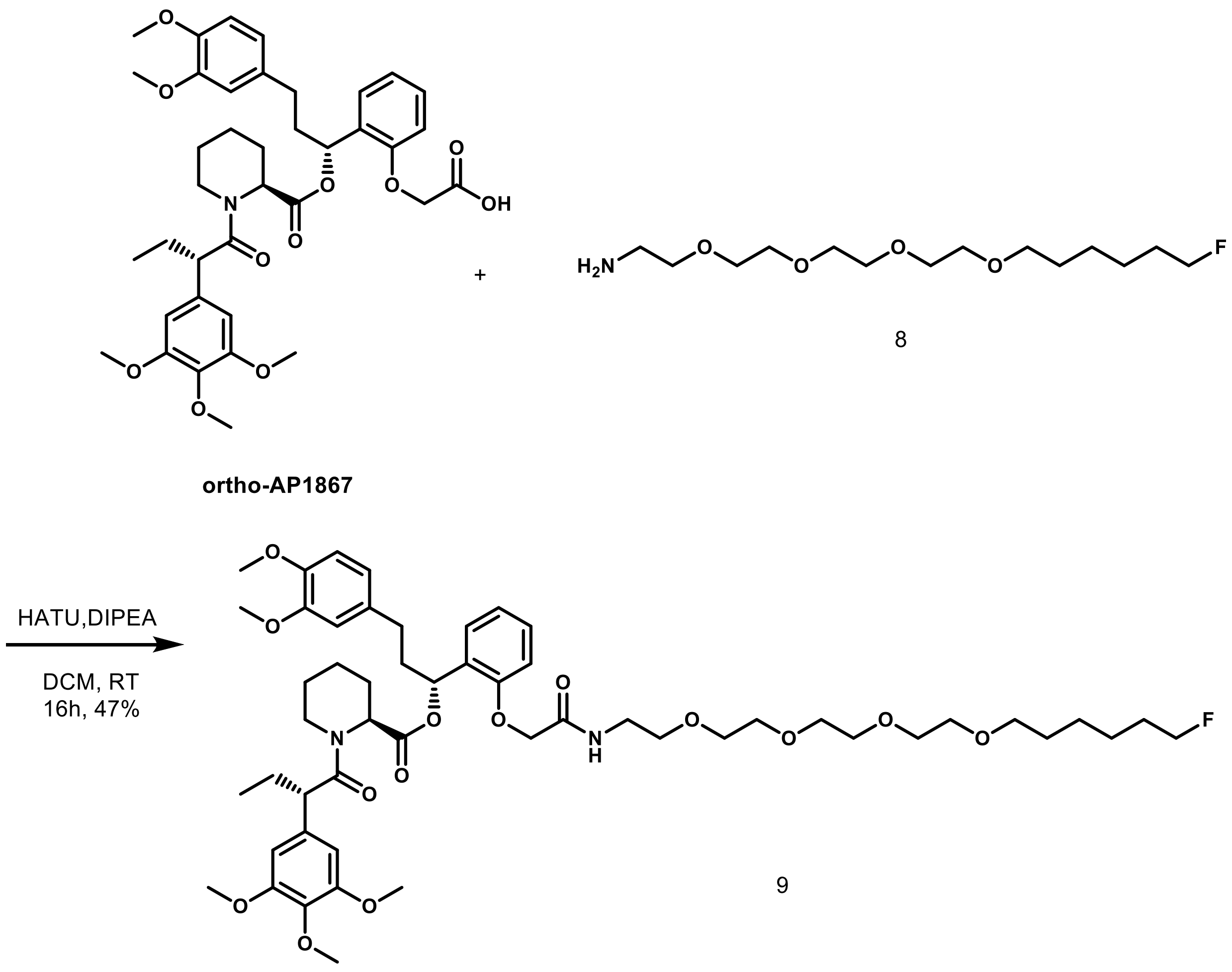

Synthesis of (R)-3-(3,4-dimethoxyphenyl)-1-(2-((21-fluoro-2-oxo-6,9,12,15-tetraoxa-3-azahenicosyl)oxy)phenyl)propyl (S)-1-((S)-2-(3,4,5trimethoxyphenyl)butanoyl)piperidine-2-carboxylate **9** (**HDPIC-Neg**)

To a solution of acid **ortho-AP1867** (0.018 g, 0.0259 mmol) in DCM (0.5 mL) were added **8** (0.05g, 0.122 mmol), DIPEA (0.05 mL, 0.259 mmol) followed by addition of HATU (0.015 g, 0.039 mmol) at RT. The reaction mixture was stirred at RT for 16 h and concentrated. The crude residue was purified by column chromatography on silica (12 g), gradient from Petroleum spirit :DCM (4:1) to 10% MeOH in Petroleum spirit : DCM (4:1). The obtained crude material (0.02 g) was further purified on HPLC RP C18 column with gradient MeCN/H_2O (5% to 95%) to give **9** (**HDPIC-Neg**) (0.012 g, 0.0122 mmol, 47%) as oil.

^{1}H NMR (500 MHz, $CDCl_3$, mixture of rotamers, only peaks of the major rotamer are reported): δ = 7.40 (t, J=5.5Hz, 1H); 7.38-7.24 (m, 1H); 7.24-7.18 (m, 1H); 7.10-7.01 (m, 1H); 6.88 (t, J=7.2 Hz, 1H); 6.86-6.76 (m, 1H); 6.73-6.66 (m, 1H); 6.69 (s, 1H); 6.49 (s, 1H); 6.44-6.25 (m, 2H); 6.16 (t, J=6.6Hz, 1H); 5.51(d, J=4.4 Hz, 1H); 4.67-4.51 (m, 4H); 4.45 (dt, J=6.2 Hz, J=47.4

Hz, 2H); 3.90-3.85 (m, 12H); 3.80 (s, 3H); 3.70 (s, 3H); 3.64-3.52 (m, 20H); 3.46 (t, J=6.5 Hz, 2H); 2.68-2.43 (m, 3H); 2.32-1.93 (m, 4H); 1.83-1.75 (m, 2H); 1.45-1.38 (m, 2H); 1.34-1.18 (m, 2H); 0.90 (t, J=7.2Hz, 3H).

^{19}F NMR: δ = - 218.13

$C_{52}H_{75}FN_2O_{14}$, expected for $[M+H]^+$ 971.53, found $[M+H]^+$ 971.5

Synthesis of PhosTAC7

Synthesized as previously reported [5], with a yield of 24%.

1. Nowak, R.P., Xiong, Y., Kirmani, N., Kalabathula, J., Donovan, K.A., Eleuteri, N.A., Yuan, J.C., and Fischer, E.S. (2021). Structure-Guided Design of a "Bump-and-Hole" Bromodomain-Based Degradation Tag. J Med Chem *64*, 11637-11650. 10.1021/acs.jmedchem.1c00958.
2. Nakanishi, M., Tahara, T., Araki, K., Shiroki, M., and Tsumagari, T. (1973). Studies on psychotropic drugs. 18. Synthesis and structure-activity relationships of 5-phenyl-1,3-dihydro-2H-thieno(2,3-e) (1,4) diazepin-2-ones. J Med Chem *16*, 214-219. 10.1021/jm00261a010.
3. Filippakopoulos, P., Qi, J., Picaud, S., Shen, Y., Smith, W.B., Fedorov, O., Morse, E.M., Keates, T., Hickman, T.T., Felletar, I., et al. (2010). Selective inhibition of BET bromodomains. Nature *468*, 1067-1073. 10.1038/nature09504.
4. Simpson, L.M., Glennie, L., Brewer, A., Zhao, J.F., Crooks, J., Shpiro, N., and Sapkota, G.P. (2022). Target protein localization and its impact on PROTAC-mediated degradation. Cell Chem Biol *29*, 1482-1504 e1487. 10.1016/j.chembiol.2022.08.004.
5. Chen, P.H., Hu, Z., An, E., Okeke, I., Zheng, S., Luo, X., Gong, A., Jaime-Figueroa, S., and Crews, C.M. (2021). Modulation of Phosphoprotein Activity by Phosphorylation Targeting Chimeras (PhosTACs). ACS Chem Biol *16*, 2808-2815. 10.1021/acschembio.1c00693.
